# Microbiology Investigation Criteria for Reporting Objectively (MICRO): a framework for the reporting and interpretation of clinical microbiology data

**DOI:** 10.1186/s12916-019-1301-1

**Published:** 2019-03-29

**Authors:** Paul Turner, Andrew Fox-Lewis, Poojan Shrestha, David A. B. Dance, Tri Wangrangsimakul, Tomas-Paul Cusack, Clare L. Ling, Jill Hopkins, Tamalee Roberts, Direk Limmathurotsakul, Ben S. Cooper, Susanna Dunachie, Catrin E. Moore, Christiane Dolecek, H. Rogier van Doorn, Philippe J. Guerin, Nicholas P. J. Day, Elizabeth A. Ashley

**Affiliations:** 1 Cambodia-Oxford Medical Research Unit, Angkor Hospital for Children, Siem Reap, Cambodia; 2 Centre for Tropical Medicine and Global Health, Nuffield Department of Medicine, University of Oxford, Oxford, UK; 3 Infectious Diseases Data Observatory, Oxford, UK; 4 Lao-Oxford-Mahosot Hospital-Wellcome Trust Research Unit, Microbiology Laboratory, Mahosot Hospital, Vientiane, Lao People’s Democratic Republic; 5 Faculty of Infectious and Tropical Diseases, London School of Hygiene and Tropical Medicine, London, UK; 6 Mahidol-Oxford Tropical Medicine Research Unit, Faculty of Tropical Medicine, Mahidol University, Bangkok, Thailand; 7 National Infection Service, Public Health England, London, UK; 8 Shoklo Malaria Research Unit, Mahidol-Oxford Tropical Medicine Research Unit, Faculty of Tropical Medicine, Mahidol University, Mae Sot, Thailand; 9 Big Data Institute, Nuffield Department of Medicine, University of Oxford, Oxford, UK; 10 Oxford University Clinical Research Unit, Hanoi, Vietnam; 11 Myanmar Oxford Clinical Research Unit, Yangon, Myanmar

**Keywords:** Antimicrobial, Susceptibility, Resistance, Microbiology, Reporting, Quality

## Abstract

**Background:**

There is a pressing need to understand better the extent and distribution of antimicrobial resistance on a global scale, to inform development of effective interventions. Collation of datasets for meta-analysis, mathematical modelling and temporo-spatial analysis is hampered by the considerable variability in clinical sampling, variable quality in laboratory practice and inconsistencies in antimicrobial susceptibility testing and reporting.

**Methods:**

The Microbiology Investigation Criteria for Reporting Objectively (MICRO) checklist was developed by an international working group of clinical and laboratory microbiologists, infectious disease physicians, epidemiologists and mathematical modellers.

**Results:**

In keeping with the STROBE checklist, but applicable to all study designs, MICRO defines items to be included in reports of studies involving human clinical microbiology data. It provides a concise and comprehensive reference for clinicians, researchers, reviewers and journals working on, critically appraising, and publishing clinical microbiology datasets.

**Conclusions:**

Implementation of the MICRO checklist will enhance the quality and scientific reporting of clinical microbiology data, increasing data utility and comparability to improve surveillance, grade data quality, facilitate meta-analyses and inform policy and interventions from local to global levels.

**Electronic supplementary material:**

The online version of this article (10.1186/s12916-019-1301-1) contains supplementary material, which is available to authorized users.

## Background

There is a global drive to combat the growing problem of antimicrobial resistance (AMR) [1, 2]. To better understand the extent of the situation, a key activity is generation and analysis of high-quality surveillance data. A specific goal of the World Health Organization (WHO) and various funding agencies is to improve AMR surveillance in low- and middle-income countries (LMICs) [3]. There has been also a concerted effort to maximise reporting and analysis of available human clinical microbiology data [4]. However, the utility of many existing AMR datasets is hampered by considerable variability in clinical sampling and laboratory practices [5], along with readily demonstrable inconsistencies in antimicrobial susceptibility testing (AST) data and reporting [6, 7]. These issues result in difficulties in data interpretation and significantly limit inter-study comparability [8]. Examples of methodological and reporting issues and problems that can arise are highlighted in Table 1.

**Table 1:** Examples of frequently occurring problems in the generation and reporting of clinical antimicrobial resistance data

Issue	Example
Failure to report key ‘bug-drug’ combinations	For both CLSI and EUCAST methods, methicillin resistance in *Staphylococcus aureus* is determined in the laboratory using cefoxitin (or historically oxacillin) resistance as a proxy [19, 20]. The cefoxitin/oxacillin result is used to infer susceptibility for all beta-lactam drugs, except those with specific activity against methicillin-resistant *S. aureus* (MRSA, i.e. ceftaroline). Thus, consistent reporting of cefoxitin/oxacillin resistance is required for unambiguous comparison of MRSA proportions between studies.
Reporting of antimicrobials tested on a subset of isolates	The issue of first- and second-line AST panels can lead to inconsistent reporting of resistance prevalence. In many instances, second-line agents (e.g. meropenem for *Escherichia coli*) are only tested on a subset of isolates (e.g. only those resistant to third-generation cephalosporins, such as ceftriaxone, or only in isolates from selected clinical specimens [19]) and reporting of incorrect overall resistance proportions can occur if inappropriate denominators are selected. For example, 100 *E. coli* isolates are tested against ceftriaxone and 10 (10%) are found to be resistant. These 10 isolates are tested subsequently against meropenem and 1 is resistant. This could be reported as 1/10 (10%) or 1/100 (1%) meropenem resistance. Neither of these percentages may be correct.
Multi-drug resistance definition and reporting	With the exception of *Mycobacterium tuberculosis* (resistance to isoniazid and rifampicin), definitions of bacterial multi-drug resistance (MDR) have been poorly defined and applied. The definition of MDR often reflects local AST selection and antibiotic availability and thus rates are difficult to compare meaningfully [21]. MDR definitions for major bacterial pathogens have been proposed recently but overall consensus is lacking for many species [10, 21, 22].
Changes to published antimicrobial susceptibility breakpoints over time	Changes in the definition of resistance can result in misleading time trends and difficulties in inter-study comparisons, if not explicitly dealt with during analysis. For example, the CLSI penicillin minimum inhibitory concentration (MIC) breakpoints for *Streptococcus pneumoniae* were updated in 2008, resulting in an increased proportion of non-meningitis isolates being reported as susceptible following the change [23].
Classification of infections by location	Hospital-acquired infections (HAI) are frequently more drug resistant than community-acquired infections (CAI) caused by the same organism [24, 25]. Failure to classify organisms by timing or location of infection can lead to significant under- or over-estimation of AMR rates.
Selection of appropriate isolates to include in analysis	Failure to account for screening specimens (e.g. swabs to determine extended spectrum beta-lactamase *Enterobacteriaceae* colonisation) and/or duplicate clinical isolates from discrete infection episodes can also result in significant overestimation of resistance [26].
Testing and reporting clinically inappropriate bug-drug combinations	The inclusion of susceptibility data for drugs with limited in vivo activity for a given pathogen (e.g. gentamicin and *Salmonella* Typhi) may lead to clinical confusion and could result in poor treatment outcomes.

To ensure that technically accurate and comparable microbiology laboratory results are produced by clinical diagnostic laboratories, various organisations and documents provide guidance on quality management (recently reviewed in [9]), antimicrobial susceptibility testing procedures (e.g. Clinical and Laboratory Standards Institute (CLSI) and European Committee on Antimicrobial Susceptibility Testing (EUCAST) guidelines) and reporting of antimicrobial susceptibility data [10, 11]. Formal accreditation of laboratory quality management by national and/or international organisations (e.g. International Standards Organisation (ISO)) is not yet feasible for laboratories in all LMICs. However, use of standard operating procedures and internal quality controls (to ensure assays are performed reliably and yield intended results) plus, if possible, participation in an external quality assurance scheme (to periodically monitor accuracy and to compare performance with other laboratories) can ensure that such laboratories perform to international quality standards [12].

Previous statements using the STROBE model (Strengthening the Reporting of Observational Studies in Epidemiology) [13] relating to infectious diseases have already been issued, including STROBE-NI (neonatal infections [14]), STROME-ID (molecular testing [15]) and STROBE-AMS (antimicrobial stewardship [16]). In particular, STROBE-NI provides some guidance on reporting of microbiological methods (checklist items 4.6–4.8) but is not comprehensive and may not be sufficiently visible to those working on non-neonatal infections. As yet there are no general recommendations for good scientific reporting of clinical microbiology methodology and results, an area that this paper aims to address.

## Methods

### Aims and use of MICRO

The proposed Microbiology Investigation Criteria for Reporting Objectively (MICRO) framework described herein is a checklist of items to be included in reports of studies involving human clinical microbiology data, originating from any region of the world, in countries of all income levels. It provides a concise and comprehensive reference for clinicians, researchers, reviewers and journals working on, critically appraising, and publishing clinical microbiology datasets. It is intended to apply to the reporting of microbiology results in any clinical study, not only observational studies, and thus, the term STROBE has not been incorporated into the name. Implementation of this checklist aims to enhance the scientific reporting of clinical microbiology data, increasing data utility to improve surveillance, grade data quality, facilitate meta-analyses and inform policy and interventions from local to global levels.

### Development of MICRO

The MICRO framework has been developed by a working group of clinical and laboratory microbiologists, infectious disease physicians, epidemiologists and mathematical modellers working in the UK and various LMICs, using an adaptation of recommended methodology [17] (Table 2). The need for a guideline was mooted during informal meetings as a result of discussions around the highly variable clarity and quality of clinical microbiology and/or AMR data in manuscripts submitted for peer-review. All pre-meeting steps were open and non-anonymised: discussions occurred by teleconference and documents were iterated and circulated electronically to the group.

**Table 2:** A summary of the MICRO framework development steps (derived from [17])

Step	Detail
Initial steps	Identify the need for a guideline
	Review the literature • Identify previous relevant guidance • Seek relevant evidence on the quality of reporting in published research articles • Identify key information related to the potential sources of bias in such studies
Pre-meeting activities	Identify participants
	Conduct informal exercise to identify key issues
	Generate a list of items for consideration at the face-to-face meeting
	Plan meeting, including preparation and of dissemination of pre-meeting materials
Face-to-face consensus meeting	Present and discuss results of pre-meeting activities
	Discuss the rationale for including items in the checklist
Post-meeting activities	Finalise the guidance statement
	Prepare manuscript for publication

### Review of published microbiology datasets from LMICs in South and South East Asia

Following on from identification of reporting problems from reviews of microbiology data from Africa [5, 8], published microbiology datasets from South and South East Asia were extracted for the ongoing AMR component of the Infectious Diseases Data Observatory-led systematic review, ‘Mapping the aetiology of non-malarial febrile illness globally in malaria-endemic regions’ (PROSPERO registration CRD42016049281). Details of the review search strategy are available at [18]. The review database was accessed on 17 June 2018, and all 177 available datasets were assessed to determine whether the following laboratory quality variables were reported (summarised in Additional file 1):

- Laboratory EQA participation
- AST methodology: scheme and version/year
- Inclusion of internal quality control information for AST testing

We further assessed each dataset to determine whether any technically inconsistent AST results were reported for the following WHO Global Antimicrobial Resistance Surveillance System (GLASS) priority pathogens: *Klebsiella pneumoniae*, *Salmonella* spp., *S. aureus* and *S. pneumoniae.* These four pathogens were selected on the basis of being important AMR organisms globally, covering both Gram-negative and Gram-positive species, with a range of demonstrable reporting problems resulting from deviations from international guidelines. The intention was to provide illustrative examples of problems frequently encountered, with potentially important consequences, rather than identify the entire range. Data extraction was performed by one author (PS), and another author (PT) verified potential reporting deviations by re-review of the relevant source manuscripts.

Laboratory quality data could be assessed for 112 studies. None of the studies included details of EQA programme participation. Four fifths (93/112; 83%) provided details of an AST guideline: CLSI in almost all cases. The year or version number was not recorded in 12/93 (13%). Use of QC organisms was documented in only 24/112 studies (21%); 14 of these mentioned specific American Type Culture Collection (ATCC) strains.

Examples of deviations from accepted AST reporting practice were detected for all organisms assessed:

- *Staphylococcus aureus****.*** Forty studies reported beta-lactam (penicillin, cephalosporin and/or carbapenem) susceptibility data for *S. aureus.* Of these, only 15 (38%) specifically included oxacillin and/or cefoxitin results. Several studies reported discordant results for 3rd generation cephalosporins and carbapenems, which would be expected to be more or less identical given the common resistance mechanism. Of note, six studies included susceptibility data for ceftazidime, an anti-pseudomonal third-generation cephalosporin with limited anti-Gram-positive activity which would not normally be tested against *S. aureus*: two reported susceptible isolates despite the absence of CLSI breakpoints.
- *Streptococcus pneumoniae.* Two studies reported testing gentamicin and identification of susceptible isolates despite there being no breakpoints defined by either EUCAST or CLSI for this ‘bug-drug’ combination. Minimum inhibitory concentration (MIC) determination is required for confirmation of reduced susceptibility to penicillin among *S. pneumoniae* isolates by both CLSI and EUCAST criteria. However, of the seven studies reporting non-susceptible pneumococci, two reported this phenotype based on oxacillin disk diffusion testing alone and one confirmed penicillin MIC in only a subset of oxacillin non-susceptible isolates.
- *Klebsiella pneumoniae****.*** Isolates were reported to be ampicillin susceptible (5–62% of isolates tested) in 5/11 (45%) studies reporting the species, despite almost universal intrinsic resistance globally and CLSI guidance to report all isolates as resistant [19].
- *Salmonella* spp. Despite absence of in vivo activity, and a specific CLSI warning against reporting, 24/76 (32%) studies reporting *Salmonella* spp. included results for gentamicin. All but one study reported susceptible isolates, ranging from 33 to 100% of isolates tested.

### Checklist development

Group discussions took place on five occasions between June 2017 and July 2018, where reporting issues, and potential checklist items, were discussed by members of the working group. The issues identified include all aspects pertaining to the unambiguous reporting of clinical microbiology data, including terminology, clinical context and sampling, organism identification and nomenclature, AST methodology, AMR definitions, handling of duplicate isolates and quality assurance (Table 3). This issue list was circulated to a wider group of clinical microbiologists, infectious diseases’ physicians, biomedical scientists (laboratory microbiologists), laboratory managers, epidemiologists and mathematical modellers (i.e. the authors of this manuscript) in advance of a meeting held in Bangkok, September 2018, where the final checklist was agreed. At the time of this meeting, the group members were all working at, or were associated with, clinical and/or research institutions in Asia (Cambodia, Indonesia, Laos, Myanmar, Nepal and Vietnam) or the UK.

**Table 3:**
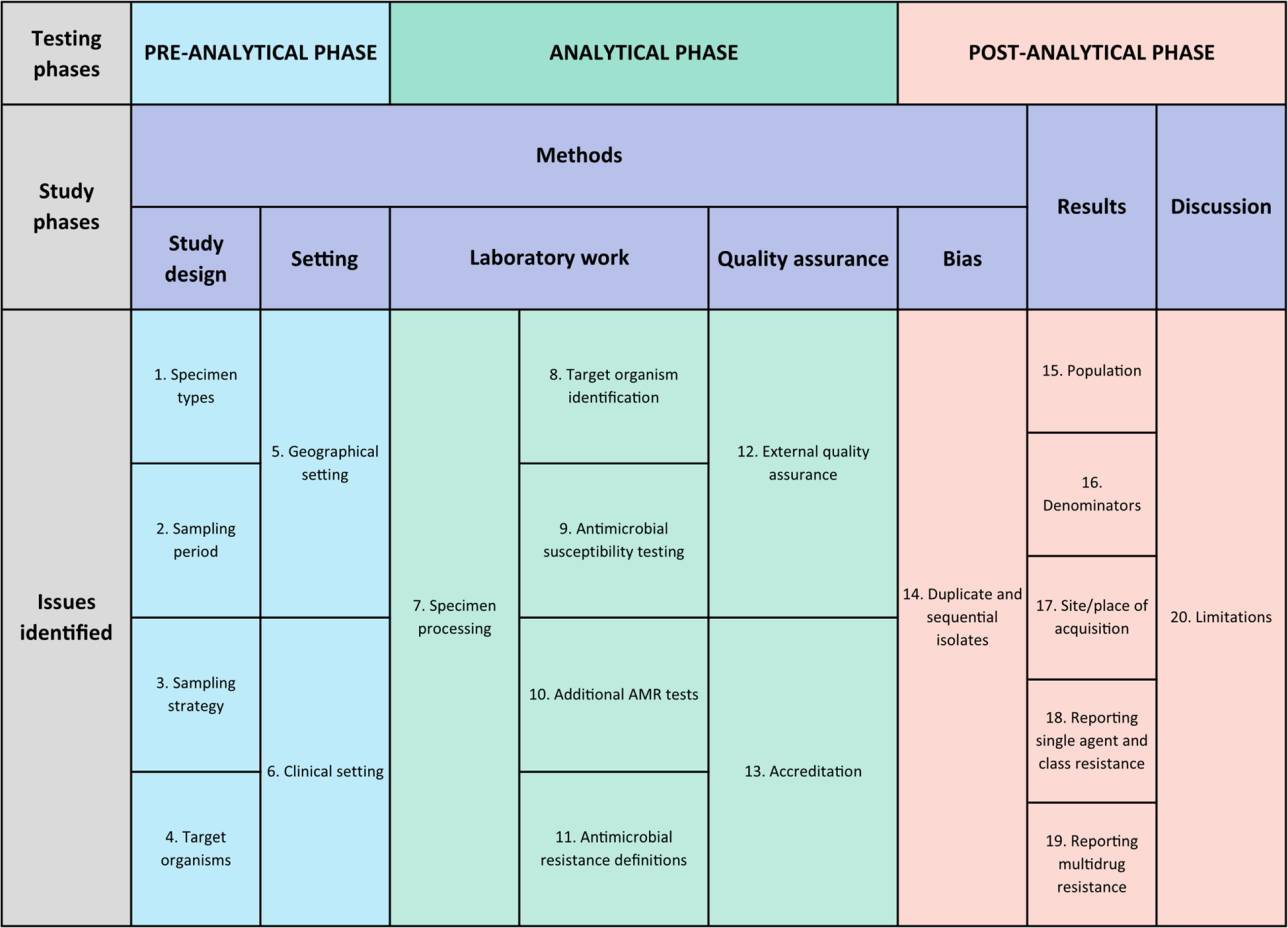
An overview of the MICRO checklist

## Results

The MICRO checklist covers important aspects of reporting of clinical microbiology data. It is expected that it will be used in conjunction with an appropriate overall study reporting statement (e.g. STROBE). Items 1–13 cover key aspects of study methodology whilst 14–20 focus on result reporting (Table 4). Core items, i.e. those that would be expected to be included in every circumstance, are indicated by an asterisk. Non-core items might be appropriately described in the manuscript Additional file 1.

**Table 4:** The MICRO framework: a checklist of items that should be addressed in reports of studies involving human clinical microbiology data

Item	Number	Recommendation
Methods		
Study design	1*	Specimen types: Describe the types of specimen included, i.e. clinical (e.g. blood cultures) or non-diagnostic surveillance (e.g. admission and other screening swabs to diagnose carriage). If specimens were obtained for diagnostic reasons, clinical syndromes should be described where possible, and specimens/isolates stratified by clinical syndrome.
	2*	Sampling period: State the collection timeframe for specimens yielding isolates for which data is reported, e.g. from MM/YY to MM/YY to be able to identify variability between seasons.
	3*	Sampling strategy: Describe the strategy for specimen collection, e.g. asymptomatic screening, sampling of all febrile patients, sampling at clinician discretion, sampling of specific patient groups and convenience sampling (e.g. use of isolates from an existing sample repository). Specify whether sampling followed routine clinical practice or was protocol driven. Classify specimens as from community-acquired (CAI) or hospital-acquired (HAI) infections. The definition of HAI used (e.g. HAI defined by specimen collection > 48 h after hospital admission) should be provided and should use ideally an international standard (e.g. US Centers for Disease Control [27, 28]).
	4	Target organisms: Explicitly state which organisms/organism groups were included in the report. Nomenclature should follow international standards (i.e. using approved genus/species names as summarised in the International Journal of Systematic and Evolutionary Microbiology). Lists of approved bacterial names can be downloaded from Prokaryotic Nomenclature Up-to-Date (https://www.dsmz.de/bacterial-diversity/prokaryotic-nomenclature-up-to-date.html) and the List of Prokaryotic Names with Standing in Nomenclature (http://www.bacterio.net/). Organisms considered contaminants should be listed, if appropriate (e.g. coagulase negative staphylococci or *Corynebacterium* spp. [29, 30]).
Setting	5*	Geographical setting: Describe the geographical distribution of specimens/patients from which isolates were obtained, at least to a country level, but preferably to a sub-national level or a geoposition.
	6*	Clinical setting: Describe the type and level of the healthcare facilities (e.g. primary, secondary, tertiary) from which specimens were obtained. If stating a microbiology laboratory, the centres served by the laboratory should be specified.
Laboratory work	7	Specimen processing: If applicable, describe specimen collection and handling, processing and sub-culture methods for all types of specimen included. For example, if reporting AST results for blood culture and cerebrospinal fluid culture isolates, the processing of these specimens by the laboratory should be briefly explained, including how specimens are sub-cultured, the media used, incubation conditions and duration. A summary of specimen processing steps (e.g. pre-processing steps, nucleic acid extraction method (if applicable), amplification platform, contamination avoidance strategy) should be provided for molecular-only workflows (e.g. to detect *Mycobacterium tuberculosis* and rifampicin resistance using the Cepheid Xpert MTB/RIF system).
	8*	Target organism identification: Details of identification methodology should be reported briefly. Where identification databases were used (e.g. bioMerieux API/bioMerieux VITEK-MS/Bruker Biotyper), the version should be specified. In general, all pathogens should be identified to species level. In the case of *Salmonella* species, organisms should be identified to at least the *S. Typhi*, *S. Paratyphi*, or non-typhoidal Salmonella (NTS) level. Strain subtyping methods should be reported according to STROME-ID [15].
	9*	Antimicrobial susceptibility testing: Describe the antimicrobial susceptibility testing methods used, internal quality control processes and their interpretation, with reference to a recognised international standard, e.g. CLSI, EUCAST. Where an international standard was followed, the specific edition(s) of guidelines used should be referenced. Deviations from standard methodology should be described, along with evidence of validation. Handling of any changes to interpretative criteria during the sampling period should be documented. State whether the raw AST data (zone diameters and/or minimum inhibitory concentrations) were re-categorised with updated breakpoints or left as-is.
	10	Additional tests performed to identify resistance mechanisms: Describe the testing methods used for adjunctive/confirmatory antimicrobial susceptibility tests, such as enzymatic/molecular assays (e.g. Xpert MTB/RIF, mecA PCR) and inducible resistance assays, with reference to a recognised international standard, where available. Where an international standard was followed, the specific edition of guidelines used should be referenced. Deviations from standard methodology should be described, along with evidence of validation.
	11*	Antimicrobial resistance definitions: Define resistance for each antimicrobial class (i.e. are isolates in the ‘intermediate’ category included within ‘susceptible’ or ‘resistant’ or analysed as a distinct category). If using the term, define MDR (e.g. ≥ 1 agent in ≥ 3 classes tested). For each organism type, an MDR test panel must be defined, consisting of the minimum panel of individual antimicrobial agents/classes against which an isolate must be tested for that isolate to be considered tested for MDR status. Antimicrobials to which an organism is intrinsically resistant cannot be part of the test panel or contribute to MDR status [10, 22].
Quality assurance	12*	External quality assurance: State whether the microbiology laboratory participates in an external quality control programme and, if so, provide scheme details. Examples include the UK National External Quality Assurance Scheme (www.ukneqasmicro.org.uk) and the American College of Pathologists External Quality Assurance/Proficiency Testing Program (https://www.cap.org/)
	13	Accreditation: State whether the laboratory is accredited through a national or international body (e.g. the International Standards Organisation, ISO) and specify which assays are covered in the accreditation.
Bias	14*	Duplicate and sequential isolates: The strategy for accounting for duplicate and sequential isolates from the same patient should be clearly detailed. Duplicate isolates are multiple isolates of the same phenotypic organism (i.e. same species and same resistance profile) from the same patient on the same date cultured either from the same clinical specimen, or from two separate clinical specimens, such as blood and CSF. Sequential isolates are isolates of the same phenotypic organism from the same patient at different dates, such as blood cultures taken on different dates. Various strategies for the handling of duplicate and sequential isolates exist [11], and the strategy used should be transparent as it will bias pooled resistance results. For example, inclusion of all isolates (the ‘all isolate strategy’) has been shown to shift pooled resistance proportions toward greater resistance, whilst inclusion of only the first isolate per patient (the ‘first isolate strategy’) or only the first isolate per infection episode (the ‘episode-based strategy’) will shift pooled results toward susceptibility.
Results		
	15*	Population: Describe the demographics of the population from which clinical specimens and subsequent isolates have been obtained, disaggregating age and gender data.
	16*	Denominators: Patient and isolate denominators should be used appropriately to ensure clarity regarding the numbers included in each analysis. Of particular importance is the reporting of resistance where first- and second-line AST panels were used (i.e. not all isolates of a particular species were tested against all agents). For drugs where only a subset of isolates were tested, reporting of a percentage without the numbers of isolates tested/resistant may be highly misleading.
	17	Site/place of acquisition: AST data from CAI and HAI should be reported and analysed separately.
	18*	Reporting resistance proportions for single agent and class resistance: Proportions of resistant isolates should be reported as number of isolates susceptible or resistant to a given antimicrobial agent/class out of actual number of isolates tested for susceptibility to that agent/class.
	19	Reporting multidrug resistance proportions: If defined, the proportion of MDR isolates should be expressed as the number of MDR isolates out of the number of isolates tested (i.e. the number undergoing the MDR test panel specific to that organism). Single agent/class resistance should be always be reported, regardless of MDR reporting.
Discussion		
Limitations	20	Discuss any reasons why bias may have been introduced into the reported data, due to patient/specimen selection, isolation of organisms, or otherwise. Consider factors which may have either introduced bias into the types of organisms isolated or the antimicrobial susceptibility profiles, e.g. receipt of antimicrobials prior to specimen collection will reduce the yield of certain species and also select for more resistant organisms.

*Core ‘must include’ items

## Discussion

The use of the MICRO checklist will result in the clinical microbiology and AST data from studies being reported in a considerably more consistent manner. In addition to its utility during preparation and peer review of study manuscripts, this checklist will be also useful to researchers when planning new studies. It will increase data quality and will reduce the publication of uninterpretable results. Data harmonisation and opportunities for sharing will be promoted. Report clarity will be improved for non-specialist readers and the prospects for meaningful comparisons between studies will be increased. Indeed, an important use of the framework would be to permit quality grading of datasets for inclusion into meta-analyses. We envisage that there might be five categories based purely on the laboratory data (Table 5), although the quality criteria could be modified depending on the intentions of the meta-analysis.

**Table 5:** An example of quality grading criteria based on the laboratory components of MICRO

Grade	Detail
A	Accreditation details provided No ID or AST errors detected
B	Accreditation details not provided EQA participation confirmed Organism ID and AST methodology completely described No ID or AST errors detected
C	Accreditation details not provided EQA participation not confirmed Organism ID and AST methodology completely described No ID or AST errors detected
D	Accreditation details not provided EQA participation not confirmed Organism ID and AST methodology partially described No ID or AST errors detected
E	Overt ID and/or AST errors detected/strongly suspected

The major limitations of this work are that we did not perform an exhaustive literature review nor carry out a formal Delphi survey to inform the checklist items. However, the reporting errors sought in the literature review for South and South East Asia were the same as those identified previously in datasets from Africa [5, 8]. Common themes arose early and agreement was reached by repeated review with the final workshop allowing consensus. The final checklist contains items that should be readily available for a quality-assured clinical microbiology laboratory service. Thus, we feel confident that the major issues are included. The checklist will be piloted within the University of Oxford Tropical Network, and we will engage actively with the EQUATOR (Enhancing the QUAlity and Transparency Of health Research) Network and relevant professional organisations to promote its use more widely. User comments will be sought following publication and implementation of the checklist. In particular, feedback from users in non-Asian settings will be valuable. It is expected that revision will be required in time. We expect that technological development will result in significant expansion of guidance on reporting of molecular-only organism identification and AST results.

## Conclusions

In summary, given the threats to human health from AMR globally, there is a pressing need to capture and model existing infection data whilst new surveillance initiatives mature sufficiently. The MICRO checklist provides a consistent and comprehensive reporting framework to ensure that interpretation and meta-analyses of such datasets are meaningful.

## Additional file

Additional file 1: Details of microbiology datasets from South and South East Asia included in the review. For each study, the antimicrobial susceptibility guideline details are summarised along with deviations from expected reporting for four bug-drug combinations: *Klebsiella pneumoniae-*ampicillin; *Salmonella* sp.*-*gentamicin; *Staphylococcus aureus-*beta-lactams; and *Streptococcus pneumoniae-*penicillin. The *Staphylococcus aureus-*beta-lactams combination includes any penicillin, cephalosporin or carbapenem apart from penicillinase-labile drugs (benzylpenicillin, phenoxymethylpenicillin, amoxicillin, ampicillin). (PDF 489 kb)

## Abbreviations

AMR: Antimicrobial resistance
AST: Antimicrobial susceptibility testing
ATCC: American Type Culture Collection
CAI: Community-acquired infection
CLSI: Clinical and Laboratory Standards Institute
CSF: Cerebrospinal fluid
EQA: External quality assurance
EQUATOR: Enhancing the QUAlity and Transparency Of health Research
EUCAST: European Committee on Antimicrobial Susceptibility Testing
GLASS: Global Antimicrobial Resistance Surveillance System
HAI: Hospital-acquired infection
ID: Identification
ISO: International Standards Organisation
LMIC: Low- and middle-income country
MDR: Multi-drug resistance
MIC: Minimum inhibitory concentration
MRSA: Methicillin-resistant *Staphylococcus aureus*
NTS: Non-typhoidal Salmonella
QC: Quality control
STROBE: Strengthening the Reporting of Observational Studies in Epidemiology
STROBE-AMS: Strengthening the Reporting of Observational Studies in Epidemiology for Antimicrobial Stewardship
STROBE-NI: Strengthening the Reporting of Observational Studies in Epidemiology for Newborn Infection
STROME-ID: Strengthening the Reporting of Molecular Epidemiology for Infectious Diseases
WHO: World Health Organization
