# Supplementary material for: Microbiology Investigation Criteria for Reporting Objectively (MICRO): a framework for the reporting and interpretation of clinical microbiology data
Source: BMC Med. 2019 Mar 29;17:70. doi: 10.1186/s12916-019-1301-1 (PMC6440102; doi:10.1186/s12916-019-1301-1)
Supplement: Supplementary file 1 — Details of microbiology datasets from South and South East Asia included in the review. For each study, the antimicrobial susceptibility guideline details are summarised along with deviations from expected reporting for four bug-drug combinations: Klebsiella pneumoniae-ampicillin; Salmonella sp.-gentamicin; Staphylococcus aureus-beta-lactams; and Streptococcus pneumoniae-penicillin. The Staphylococcus aureus-beta-lactams combination includes any penicillin, cephalosporin or carbapenem apart from penicillinase-labile drugs (benzylpenicillin, phenoxymethylpenicillin, amoxicillin, ampicillin). (PDF 489 kb) [file 12916_2019_1301_MOESM1_ESM.pdf]

Supplementary Material

Microbiology Investigation Criteria for Reporting Objectively (MICRO): a framework for the reporting and interpretation of clinical microbiology data

Paul Turner, Andrew Fox-Lewis, Poojan Shrestha, David A.B. Dance, Tri Wangrangsimakul, Tomas-Paul Cusack, Clare L. Ling, Jill Hopkins, Tamalee Roberts, Direk Limmathurotsakul, Ben S. Cooper, Susanna Dunachie, Catrin E. Moore, Christiane Dolecek, H. Rogier van Doorn, Philippe J. Guerin, Nicholas P.J. Day Elizabeth A. Ashley

Details of microbiology datasets from South and South East Asia included in the review

For each study, the antimicrobial susceptibility guideline details are summarised along with deviations from expected reporting for four bug-drug combinations: *Klebsiella pneumoniae* – ampicillin; *Salmonella* sp. – gentamicin; *Staphylococcus aureus* – beta-lactams; *Streptococcus pneumoniae* – penicillin. The *Staphylococcus aureus* – beta-lactams combination includes any penicillin, cephalosporin, or carbapenem apart from penicillinase-labile drugs (benzylpenicillin, phenoxymethylpenicillin, amoxicillin, ampicillin).

AST: antimicrobial susceptibility testing; BSAC: British Society for Antimicrobial Chemotherapy; CLSI (NCCLS): Clinical and Laboratory Standards Institute (National Committee for Clinical Laboratory Standards); EUCAST: European Committee on Antimicrobial Susceptibility Testing; NICD: National Institute for Communicable Diseases; WHO: World Health Organization.

First author	Manuscript title	Publication year	Stated AST guideline	Guideline version	Target organism	Target antimicrobial	Problem identified	Ref.
Abdullah FE	Enteric fever in Karachi: current antibiotic susceptibility of *Salmonellae* isolates	2012	Not stated					(1)
Achana V	Acute *Pseudomonas pseudomallei* pneumonia and septicemia following aspiration of contaminated water: a case report	1985						(2)
Acharya D	Antibiotic susceptibility pattern and the indicator of decreased ciprofloxacin susceptibility of *Salmonella enterica* serovar Typhi isolated from Dhulikhel Hospital, Nepal	2012	Not stated					(3)
Agnihotri N	Antimicrobial susceptibility of isolates from neonatal septicemia	2004	CLSI (NCCLS)	2000	*Staphylococcus aureus*	Beta-lactams	Does not report oxacillin / cefoxitin	(4)
Al-Taiar A	Neonatal infections in China, Malaysia, Hong Kong and Thailand	2013	Not stated					(5)
Amatya NM	Etiological agents of bacteraemia and antibiotic susceptibility pattern in Kathmandu Model Hospital	2007	CLSI (NCCLS)	Not stated				(6)
Anand AC	The anatomy of an epidemic (the final report on an epidemic of multidrug resistant enteric fever in eastern India)	1993			*Salmonellae*	Gentamicin	Reports result (and susceptible isolates)	(7)
Anderson M	Epidemiology of bacteremia in young hospitalized infants in Vientiane, Laos, 2000-2011	2014	CLSI (NCCLS)	2010	*Klebsiella pneumoniae*	Ampicillin	None	(8)
					Staphylococcus aureus	Beta-lactams	None	
Anekthananon T	Community-acquired *Klebsiella* bacteremia	2001	Not stated					(9)
Anjum P	Fluroquinolone resistance in typhoidal *Salmonella* and its detection by nalidixic acid disc diffusion	2004	CLSI (NCCLS)	Not stated				(10)

First author	Manuscript title	Publication year	Stated AST guideline	Guideline version	Target organism	Target antimicrobial	Problem identified	Ref.
Anthony B	Spectrum of melioidosis in the suburbs of Mangalore, S west coast of India	2010	CLSI (NCCLS)	2005				(11)
Anunnatsiri S	Febrile neutropenia: a retrospective study in Srinagarind Hospital	1998	Not stated		*Staphylococcus aureus*	Beta-lactams	None	(12)
Arafat MY	Current Pattern in Antimicrobial Susceptibility in Enteric Fever In A Private Medical College	2014			*Salmonellae*	Gentamicin	Reports result (and susceptible isolates)	(13)
Arifeen SE	Invasive pneumococcal disease among children in rural Bangladesh: results from a population-based surveillance	2009			*Streptococcus pneumoniae*	Penicillin	None	(14)
Ariffin H	Septicaemia in paediatric cancer patients: a 5-year surveillance study in university hospital, Kuala Lumpur, Malaysia	1997	Not stated		*Staphylococcus aureus*	Beta-lactams	Reports methicillin but not oxacillin / cefoxitin	(15)
Arora RK	Multidrug resistant typhoid fever: study of an outbreak in Calcutta	1992			*Salmonellae*	Gentamicin	Reports result (and susceptible isolates)	(16)
Arora S	Changing susceptibility patterns of nonfermenting Gram-negative bacilli	2012	CLSI (NCCLS)	2010				(17)
Aurangzeb B	Neonatal sepsis in hospital-born babies: bacterial isolates and antibiotic susceptibility patterns	2003						(18)
Ayyagari A	Outbreak of typhoid fever due to multiresistant *Salmonella typhi* in northern India - a preliminary report	1991						(19)
Bajracharya BL	Clinical profile and antibiotics response in typhoid fever	2006						(20)
Baker S	Fatal wound infection caused by *Chromobacterium violaceum* in Ho Chi Minh City, Vietnam	2008	CLSI (NCCLS)	2007				(21)
Banerjee M	Outbreak of neonatal septicemia with multidrug resistant *Klebsiella pneumoniae*	1993			*Klebsiella pneumoniae*	Ampicillin	None	(22)
Bankar S	Study of ascitic fluid for diagnosis of spontaneous bacterial peritonitis (SBP) in adult patients with cirrhosis	2014	CLSI (NCCLS)	2013	*Staphylococcus aureus*	Beta-lactams	None	(23)
Baskaran ND	Bacteremia in patients with febrile neutropenia after chemotherapy at a university medical center in Malaysia	2007	CLSI (NCCLS)	2004	*Staphylococcus aureus*	Beta-lactams	Reports methicillin but not oxacillin / cefoxitin	(24)
Behera B	Ceftazidime resistance in *Burkholderia pseudomallei*: first report from India	2012	Not stated (but states MIC cut-offs)					(25)
Bhagawati G	Study on isolates of acute meningitis in a tertiary care centre in Assam	2014	CLSI (NCCLS)	2010	*Staphylococcus aureus*	Beta-lactams	Does not report oxacillin / cefoxitin	(26)
					Streptococcus pneumoniae	Gentamicin / Penicillin	Reports isolates susceptible to gentamicin and penicillin non-	

First author	Manuscript title	Publication year	Stated AST guideline	Guideline version	Target organism	Target antimicrobial	Problem identified	Ref.
							susceptibility on basis of disk testing only	
Bhagra S	Antibiotic susceptibility pattern of *Salmonella enterica* serovar Typhi and Paratyphi A from North India: The changing scenario	2014	CLSI (NCCLS)	2012				(27)
Bhaskar MM	Changing paradigm of enterococcal infections	2015	CLSI (NCCLS)	2013				(28)
Bhat KG	Neonatal bacteremia due to high level aminoglycoside resistant (HLAR) enterococci	1997	CLSI (NCCLS)	1990				(29)
Bhattacharya SS	Occurrence of *Salmonella typhi* infection in Rourkela, Orissa	2000	WHO	1987	*Salmonellae*	Gentamicin	Reports result (and susceptible isolates)	(30)
Bhattacharya SS	Occurrence & antibiogram of *Salmonella* Typhi & *S.* Paratyphi A isolated from Rourkela, Orissa	2011	CLSI (NCCLS)	2006	*Salmonellae*	Gentamicin	Reports result (and susceptible isolates)	(31)
Bindu D	Bacterial profile and antibiotic susceptibility pattern of blood culture isolates from pediatric age group attending a tertiary care centre	2013			*Staphylococcus aureus*	Beta-lactams	Does not report oxacillin / cefoxitin	(32)
Boonyagars L	Meningitis and spondylodiscitis due to *Streptococcus suis*	2010	Not stated					(33)
Butt T	Central venous catheter-related bloodstream infections in cancer patients	2004	CLSI (NCCLS)	2000	*Staphylococcus aureus*	Beta-lactams	None	(34)
Butt T	Changing trends in drug resistance among typhoid *salmonellae* in Rawalpindi, Pakistan	2005	CLSI (NCCLS)	2000				(35)
Capoor MR	*Salmonella enterica* serovar Typhi: molecular analysis of strains with decreased susceptibility and resistant to ciprofloxacin in India from 2001-2003	2007	CLSI (NCCLS)	2005				(36)
Chaikittisuk N	*Salmonella* bacteremia in children: a changing pattern of antibiotic resistance	2000	CLSI (NCCLS)	Not stated	*Salmonellae*	Gentamicin	Reports result (and susceptible isolates)	(37)
Chande C	Change in antimicrobial resistance pattern of *Salmonella* Typhi in central India	2002	CLSI (NCCLS)	1997	*Salmonellae*	Gentamicin	Reports result (and susceptible isolates)	(38)
Chim H	*Aeromonas* infection in critically ill burn patients	2007	CLSI (NCCLS)	Not stated				(39)
Chlebicki MP	First outbreak of colonization and infection with vancomycin-resistant *Enterococcus faecium* in a tertiary care hospital in Singapore	2006						(40)
Chong CY	Invasive pneumococcal disease in Singapore children	2009			*Streptococcus pneumoniae*	Penicillin	None	(41)

First author	Manuscript title	Publication year	Stated AST guideline	Guideline version	Target organism	Target antimicrobial	Problem identified	Ref.
Choudhary A	Antimicrobial susceptibility of *Salmonella enterica* serovars in a tertiary care hospital in southern India	2013	CLSI (NCCLS)	2006				(42)
Deris ZZ	The prevalence and risk factors of nosocomial *Acinetobacter* blood stream infections in tertiary teaching hospital in north-eastern Malaysia	2009						(43)
Dhanashree B	Antibiotic susceptibility profile of *Salmonella enterica* serovars: trend over three years showing re-emergence of chloramphenicol sensitivity and rare serovars	2007	CLSI (NCCLS)	2005				(44)
Duggal S	Recent outbreak of meningococcal meningitis--a microbiological study with brief review of literature	2007	CLSI (NCCLS) / NICD	2005				(45)
Duggal S	Etiology and susceptibility of blood stream infections in a Referral Hospital in North Delhi: A one year study	2014	CLSI (NCCLS)	2007				(46)
Dutta S	Rollback of *Salmonella enterica* serotype typhi resistance to chloramphenicol and other antimicrobials in Kolkata, India	2005						(47)
Easow JM	Blood stream infections among febrile patients attending a teaching hospital in western region of Nepal	2010	CLSI (NCCLS)	2005	*Salmonellae*	Gentamicin	Reports result (and susceptible isolates)	(48)
					Staphylococcus aureus	Beta-lactams	None	
Emary K	Enteric fever in Cambodian children is dominated by multidrug-resistant H58 *Salmonella enterica* serovar Typhi with intermediate susceptibility to ciprofloxacin	2012	CLSI (NCCLS)	2012				(49)
Geetha VK	Plasmid-mediated quinolone resistance in typhoidal *Salmonellae*: a preliminary report from South India	2014	CLSI (NCCLS)	2012				(50)
Geethu M	Antimicrobial susceptibility trends among viridans streptococci isolates from cases of endocarditis from 2007-2009	2010	CLSI (NCCLS)	Not stated				(51)
Gopal Katherason S	Prospective surveillance of nosocomial device-associated bacteremia in three adult intensive units in Malaysia	2010	Not stated		*Staphylococcus aureus*	Beta-lactams	Does not report oxacillin / cefoxitin; reports ceftazidime	(52)
Gupta P	Clinical profile of *Klebsiella* septicemia in neonates	1993						(53)

First author	Manuscript title	Publication year	Stated AST guideline	Guideline version	Target organism	Target antimicrobial	Problem identified	Ref.
Gupta V	An increase in enteric fever cases due to *Salmonella* Paratyphi A in & around Chandigarh	2009	CLSI (NCCLS)	2006				(54)
Gyawali N	Bacteriological profile and antibiogram of neonatal septicemia	2013	CLSI (NCCLS)	2006	*Staphylococcus aureus*	Beta-lactams	Does not report oxacillin / cefoxitin	(55)
Habib AG	Community-acquired *Klebsiella pneumoniae* central nervous system infections in adults in Singapore	2003			*Klebsiella pneumoniae*	Ampicillin	None	(56)
Halder D	Neonatal meningitis and septicaemia caused by *Burkholderia pseudomallei*	1998	Not stated					(57)
Haque SM	Identification of bacterial isolates in neonatal sepsis and their antimicrobial susceptibility	2014	CLSI (NCCLS)	Not stated	*Klebsiella pneumoniae*	Ampicillin	None	(58)
					Staphylococcus aureus	Beta-lactams	None	
Heng BH	Epidemiological surveillance of melioidosis in Singapore	1998						(59)
Hoa NT	Community-acquired septicaemia in southern Vietnam: the importance of multidrug-resistant *Salmonella typhi*	1998	CLSI (NCCLS)	1993	*Staphylococcus aureus*	Beta-lactams	Reports methicillin but not oxacillin / cefoxitin	(60)
Hsu LY	Six cases of daptomycin-non-susceptible *Staphylococcus aureus* bacteraemia in Singapore	2010	Not stated					(61)
Imran MN	Early predictors of mortality in pneumococcal bacteraemia	2005	CLSI (NCCLS)	1990	*Streptococcus pneumoniae*	Penicillin	None	(62)
Iqbal Hossain M	*Acinetobacter* bacteremia in patients with diarrhoeal disease	1998						(63)
Jain N	*Globicatella sanguinis* meningitis in a post head trauma patient: first case report from Asia	2012	CLSI (NCCLS)	2009				(64)
Jesudason MV	Septicaemic melioidosis in a tertiary care hospital in south India	2003	Not stated					(65)
Jitsurong S	Prevalence of extended-spectrum beta-lactamases (ESBLs) produced in blood isolates of gram-negative bacteria in a teaching hospital in Southern Thailand	2006	CLSI (NCCLS)	2000	*Klebsiella pneumoniae*	Ampicillin	None	(66)
Kabra SK	Multidrug-resistant typhoid fever	2000	Not stated		*Salmonellae*	Gentamicin	Reports result (and susceptible isolates)	(67)
Kamili MA	Multiple drug resistant typhoid fever outbreak in Kashmir Valley	1993						(68)
Kapoor L	Microbiological profile of neonatal septicemia in a pediatric care hospital in Delhi	2005	Stokes	Not applicable	*Staphylococcus aureus*	Beta-lactams	Does not report oxacillin / cefoxitin	(69)
Karki AB	Higher nalidixic acid resistance pattern of *Salmonella* isolates	2013	Not stated					(70)

First author	Manuscript title	Publication year	Stated AST guideline	Guideline version	Target organism	Target antimicrobial	Problem identified	Ref.
	from enteric fever patients in Kathmandu Model Hospital, Nepal							
Karki S	Bacteriological analysis and antibiotic sensitivity pattern of blood culture isolates in Kanti Children Hospital	2010			*Klebsiella pneumoniae*	Ampicillin	None	(71)
					Streptococcus pneumoniae	Penicillin	None	
Karthikeyan G	Neonatal sepsis: *Staphylococcus aureus* as the predominant pathogen	2001			*Klebsiella pneumoniae*	Ampicillin	Reports susceptible isolates	(72)
					Staphylococcus aureus	Beta-lactams	Does not report oxacillin / cefoxitin	
Kasper MR	Emergence of multidrug-resistant *Salmonella enterica* servoar Typhi with reduced susceptibility to fluoroquinolones in Cambodia	2010	CLSI (NCCLS)	2009				(73)
Khairulddin NYN	Epidemiology of *Haemophilus influenzae* invasive disease in hospitalised Kelantanese children, 1985-1994	1999	Stokes	Not applicable				(74)
Khan MI	Non-typhoidal *Salmonella* rates in febrile children at sites in five Asian countries	2010						(75)
Komolpis P	*Salmonella* bacteremia: serotype distribution and antimicrobial susceptibility during 1991-1995	1999	Not stated		*Salmonellae*	Gentamicin	Reports result (and susceptible isolates)	(76)
Kruse AY	Neonatal bloodstream infections in a pediatric hospital in Vietnam: a cohort study	2013	CLSI (NCCLS)	2008	*Staphylococcus aureus*	Beta-lactams	None	(77)
Kumar A	An outbreak of multidrug resistant *Salmonella typhimurium* in a nursery	1995			*Salmonellae*	Gentamicin	Reports result (and susceptible isolates)	(78)
Kumarasamy K	Report of a *Salmonella enterica* serovar Typhi isolate from India producing CMY-2 AmpC peta-lactamase	2012	CLSI (NCCLS) / EUCAST	2011 / 2011	*Salmonellae*	Gentamicin	Reports result (and susceptible isolates)	(79)
Lee WS	Antimicrobial susceptibility and distribution of non-typhoidal *Salmonella* serovars isolated in Malaysian children	2003	CLSI (NCCLS)	Not stated	*Salmonellae*	Gentamicin	Reports result (and susceptible isolates)	(80)
Lim NL	Bacteraemic infections in a neonatal intensive care unit--a nine-month survey	1995						(81)
Litzow JM	High frequency of multidrug-resistant gram-negative rods in two neonatal intensive care units in the Philippines	2009	Not stated					(82)
Lulitanond A	Virulence genes and genotypes of *Staphylococcus aureus* from blood of Thai patients	2015	CLSI (NCCLS)	2010	*Staphylococcus aureus*	Beta-lactams	None	(83)
Lupisan SP	Invasive bacterial infections of children in a rural province in the central Philippines	2000	CLSI (NCCLS)	Not stated	*Streptococcus pneumoniae*	Penicillin	None	(84)

First author	Manuscript title	Publication year	Stated AST guideline	Guideline version	Target organism	Target antimicrobial	Problem identified	Ref.
Manchanda V	Treatment of enteric fever in children on the basis of current trends of antimicrobial susceptibility of *Salmonella enterica* serovar Typhi and Paratyphi A	2006	CLSI (NCCLS)	2004	*Salmonellae*	Gentamicin	Reports result (and susceptible isolates)	(85)
Maskey AP	*Salmonella enterica* serovar Paratyphi A and *S. enterica* serovar Typhi cause indistinguishable clinical syndromes in Kathmandu, Nepal	2006	CLSI (NCCLS)	2003				(86)
Maskey AP	Emerging trends in enteric fever in Nepal: 9124 cases confirmed by blood culture 1993-2003	2008	CLSI (NCCLS)	2000				(87)
Mehar V	Neonatal sepsis in a tertiary care center in central India: microbiological profile, antimicrobial sensitivity pattern and outcome	2013			*Staphylococcus aureus*	Beta-lactams	Does not report oxacillin / cefoxitin but AST done by VITEK (MRSA reported incorrectly)	(88)
Mehar V	*Pantoea dispersa*: an unusual cause of neonatal sepsis	2013						(89)
Mishra S	Multidrug resistant typhoid fever: therapeutic considerations	1992			*Salmonellae*	Gentamicin	Reports result (and susceptible isolates)	(90)
Mohanty S	Antibiogram pattern and seasonality of *Salmonella* serotypes in a North Indian tertiary care hospital	2006	CLSI (NCCLS)	2004				(91)
Mohanty S	Bacteriology of parapneumonic pleural effusions in an Indian hospital	2007	CLSI (NCCLS)	Not stated	*Staphylococcus aureus*	Beta-lactams	Reports methicillin but not oxacillin / cefoxitin	(92)
					Streptococcus pneumoniae	Penicillin		
Mortlock S	Bacteraemia among patients attending a cancer hospital in Lahore, Pakistan	2000	CLSI (NCCLS)	1988	*Staphylococcus aureus*	Beta-lactams	Reports methicillin but not oxacillin / cefoxitin	(93)
Muhammad Z	Neonatal sepsis: causative bacteria and their resistance to antibiotics	2010			*Staphylococcus aureus*	Beta-lactams	Does not report oxacillin / cefoxitin; reports ceftazidime	(94)
Muley VA	Bacteriological profile of neonatal septicemia in a tertiary care hospital from Western India	2015	CLSI (NCCLS)	2011	*Klebsiella pneumoniae*	Ampicillin	Reports susceptible isolates	(95)
					Staphylococcus aureus	Beta-lactams	Does not report oxacillin / cefoxitin	
Murdoch DR	The etiology of febrile illness in adults presenting to Patan Hospital in Kathmandu, Nepal	2004	CLSI (NCCLS)	2000				(96)
Naheed A	Burden of typhoid and paratyphoid fever in a densely populated urban community, Dhaka, Bangladesh	2010	CLSI (NCCLS)	2006				(97)
Najeeb S	Causative bacteria and antibiotic resistance in neonatal sepsis	2012			*Staphylococcus aureus*	Beta-lactams	Does not report oxacillin / cefoxitin; reports ceftazidime	(98)
Nakwan N	Clinical features, risk factors, and outcome of carbapenem-resistant	2012	CLSI (NCCLS)	2010				(99)

First author	Manuscript title	Publication year	Stated AST guideline	Guideline version	Target organism	Target antimicrobial	Problem identified	Ref.
	Acinetobacter baumannii bacteremia in a Thai neonatal intensive care unit							
Nandhakumar B	Penicillin-resistant viridans group streptococci from blood cultures of infective endocarditis patients in South India	2008	CLSI (NCCLS)	2007				(100)
Nema S	Antibiogram study over bacterial isolates from cases of bacteraemias	1996			*Salmonellae*	Gentamicin	Reports result (and susceptible isolates)	(101)
Neopane A	Changing spectrum of antibiotic sensitivity in enteric fever	2008						(102)
Nickerson EK	*Staphylococcus aureus* bacteraemia in a tropical setting: patient outcome and impact of antibiotic resistance	2009			*Staphylococcus aureus*	Beta-lactams	None	(103)
Ochiai RL	A study of typhoid fever in five Asia countries: disease burden and implications for controls	2008	Not stated					(104)
Ong C WM	Severe community-acquired *Acinetobacter baumannii* pneumonia: an emerging highly lethal infectious disease in the Asia-Pacific	2009	CLSI (NCCLS)	Not stated				(105)
Pais M	Neonatal sepsis, bacterial isolates and antibiotic susceptibility patterns among neonates	2012			*Staphylococcus aureus*	Beta-lactams	Does not report oxacillin / cefoxitin; reports ceftazidime	(106)
Palash S	Emerging resistance of non-fermenting gram negative bacilli in a tertiary care centre	2011	CLSI (NCCLS)	2007				(107)
Pancharoen C	Meningococcal infection in children	1998	CLSI (NCCLS)	Not stated but did define MIC breakpoint s				(108)
Pandit V	Study of clinical profile and antibiotic sensitivity in paratyphoid fever cases admitted at teaching hospital in South India	2012						(109)
Parveen RM	Extended-spectrum beta-lactamase producing *Klebsiella pneumoniae* from blood cultures in Puducherry, India	2011	CLSI (NCCLS)	2007				(110)
Pathengay A	Clinical and microbiologic review of culture-proven endophthalmitis caused by multidrug-resistant bacteria in patients seen at a tertiary eye care center in southern India	2011						(111)
Phan LT	Genetic and phenotypic characterization of *Haemophilus influenzae* type b isolated from	2006						(112)

First author	Manuscript title	Publication year	Stated AST guideline	Guideline version	Target organism	Target antimicrobial	Problem identified	Ref.
	children with meningitis and their family members in Vietnam							
Phe T	Does HIV status affect the aetiology, bacterial resistance patterns and recommended empiric antibiotic treatment in adult patients with bloodstream infection in Cambodia?	2013	CLSI (NCCLS)	2012	*Staphylococcus aureus*	Beta-lactams	None	(113)
Phetsouvanh R	Causes of community-acquired bacteremia and patterns of antimicrobial resistance in Vientiane, Laos	2006	CLSI (NCCLS)	1998	*Klebsiella pneumoniae*	Ampicillin	Reports susceptible isolates	(114)
					Staphylococcus aureus	Beta-lactams	Does not report oxacillin / cefoxitin	
					Streptococcus pneumoniae	Penicillin	Incomplete testing of oxacillin non-susceptible isolates (penicillin MIC only done on a subset)	
Pocock JM	Septic arthritis of the hip in a Cambodian child caused by multidrug-resistant *Salmonella enterica* serovar Typhi with intermediate susceptibility to ciprofloxacin treated with ceftriaxone and azithromycin	2014						(115)
Pokharel BM	Multidrug-resistant and extended-spectrum beta-lactamase (ESBL)-producing *Salmonella enterica* (serotypes Typhi and Paratyphi A) from blood isolates in Nepal: surveillance of resistance and a search for newer alternatives	2006	CLSI (NCCLS)	2004				(116)
Pokharel P	Study of enteric fever and antibiogram of *Salmonella* isolates at a teaching hospital in Kathmandu Valley	2009			*Salmonellae*	Gentamicin	Reports result (and susceptible isolates)	(117)
Porter KA	*Acinetobacter* bacteraemia in Thailand: evidence for infections outside the hospital setting	2014	CLSI (NCCLS)	2012				(118)
Pradhan R	Bloodstream infection among children presenting to a general hospital outpatient clinic in urban Nepal	2012	CLSI (NCCLS)	2006	*Streptococcus pneumoniae*	Penicillin	None	(119)
Punpanich W	Invasive salmonellosis in urban Thai children: a ten-year review	2012						(120)
Qamar FN	A three-year review of antimicrobial resistance of *Salmonella enterica* serovars Typhi and Paratyphi A in Pakistan	2014	CLSI (NCCLS)	2009				(121)
Qamar MU	Metallo-beta-lactamase producing *Enterobacter cloacae*: an emerging threat in neonates	2014	CLSI (NCCLS)	2009				(122)
Rai S	Rationale of azithromycin prescribing practices for enteric fever in India	2012	CLSI (NCCLS) / BSAC	2009 / 2010				(123)

First author	Manuscript title	Publication year	Stated AST guideline	Guideline version	Target organism	Target antimicrobial	Problem identified	Ref.
Rao RS	A study of drug resistance among *Salmonella typhi* and *Salmonella paratyphi A* in an endemic area, 1977-79	1981						(124)
Raza S	Antimicrobial susceptibility patterns of *Salmonella typhi* and *Salmonella paratyphi A* in a tertiary care hospital	2012	CLSI (NCCLS)	1997	*Salmonellae*	Gentamicin	Reports result (and susceptible isolates)	(125)
Renuka K	Reduced susceptibility to ciprofloxacin and gyra gene mutation in North Indian strains of *Salmonella enterica* serotype Typhi and serotype Paratyphi A	2004	CLSI (NCCLS)	2002				(126)
Rhodes J	Pneumococcal bacteremia requiring hospitalization in rural Thailand: an update on incidence, clinical characteristics, serotype distribution, and antimicrobial susceptibility, 2005-2010	2013	CLSI (NCCLS)	2008	*Streptococcus pneumoniae*	Penicillin	None	(127)
Roy S	Tigecycline susceptibility in *Klebsiella pneumoniae* and *Escherichia coli* causing neonatal septicaemia (2007-10) and role of an efflux pump in tigecycline non-susceptibility	2013	CLSI (NCCLS) / EUCAST	2008 / 2011				(128)
Sharma NP	A hospital-based study of bloodstream infections in febrile patients in Dhulikhel Hospital Kathmandu University Teaching Hospital, Nepal	2006						(129)
Sharma P	*Staphylococcus aureus* - the predominant pathogen in the neonatal ICU of a tertiary care hospital in Amritsar, India	2013	CLSI (NCCLS)	2007	*Staphylococcus aureus*	Beta-lactams	None	(130)
Shaw P	Microbial array and antibiotic sensitivity pattern of catheter related blood-stream infection at a tertiary care hospital in South India	2012						(131)
Sheikh A	*Salmonella enterica* serovar Typhi-specific immunoglobulin A antibody responses in plasma and antibody in lymphocyte supernatant specimens in Bangladeshi patients with suspected typhoid fever	2009			*Salmonellae*	Gentamicin	Reports result (and susceptible isolates)	(132)
Sheikh AN	Neonatal sepsis: An evaluation of bacteriological spectrum, antibiotic susceptibilities and prognostic predictors at Civil Hospital, Karachi	2014			*Staphylococcus aureus*	Beta-lactams	Does not report oxacillin / cefoxitin	(133)
Sheikh SO	High rate of non-susceptibility to metronidazole and clindamycin in	2015	CLSI (NCCLS)	2012				(134)

First author	Manuscript title	Publication year	Stated AST guideline	Guideline version	Target organism	Target antimicrobial	Problem identified	Ref.
	anaerobic isolates: data from a clinical laboratory from Karachi, Pakistan							
Shenoy S	Penicillin resistant *Streptococcus pneumoniae*	2003	CLSI (NCCLS)	1995	*Streptococcus pneumoniae*	Oxacillin	None	(135)
Shivaprakasha S	Late prosthetic valve endocarditis due to *Cardiobacterium hominis*, an unusual complication	2007						(136)
Shivaprakasha S	Cerebral artery mycotic aneurysm associated with *Erysipelothrix rhusiopathiae* endocarditis	2007						(137)
Shoma S	Rapid detection of *Haemophilus influenzae* type b in Bangladeshi children with pneumonia and meningitis by PCR and analysis of antimicrobial resistance	2001						(138)
Shrestha RK	Bacteriological study of neonatal sepsis and antibiotic susceptibility pattern of isolates in Kathmandu, Nepal	2013	CLSI (NCCLS)	2006	*Klebsiella pneumoniae*	Ampicillin	Reports susceptible isolates	(139)
					Salmonellae	Gentamicin	Reports result (and susceptible isolates)	
					Staphylococcus aureus	Beta-lactams	Does not report oxacillin / cefoxitin	
Shrestha S	Antibiotic resistance pattern of bacterial isolates in neonatal care unit	2010			*Staphylococcus aureus*	Beta-lactams	Does not report oxacillin / cefoxitin	(140)
Shrestha S	Bacterial isolates and its antibiotic susceptibility pattern in NICU	2013			*Staphylococcus aureus*	Beta-lactams	Does not report oxacillin / cefoxitin	(141)
Shwe TN	Blood culture isolates from children admitted to Medical Unit III, Yangon Children's Hospital, 1998	2002			*Salmonellae*	Gentamicin	Reports result (and susceptible isolates)	(142)
					Staphylococcus aureus	Beta-lactams	Does not report oxacillin / cefoxitin	
Singh SD	Enteric fever in Children at Dhulikhel Hospital	2012			*Salmonellae*	Gentamicin	Reports result (and susceptible isolates)	(143)
Singh UK	*Salmonella typhi* infections and effect of fluroquinolones and third generation cephalosporins in clinical outcome	2011						(144)
Singhal L	Trends in antimicrobial susceptibility of *Salmonella* Typhi from North India (2001-2012)	2014	CLSI (NCCLS)	2012				(145)
Singhi S	Nosocomial bloodstream infection in a pediatric intensive care unit	2008			*Staphylococcus aureus*	Beta-lactams	Reports methicillin but not oxacillin / cefoxitin	(146)
Singla N	Outbreak of *Salmonella* Typhi enteric fever in sub-urban area of North India: a public health perspective	2013	CLSI (NCCLS)	2009				(147)
Srifuengfung S	*Salmonella* meningitis and antimicrobial susceptibilities	2005	CLSI (NCCLS)	2003				(148)
Srifuengfung S	*Roseomonas gilardii* subsp *rosea*, a pink bacterium associated with bacteremia: the first case in Thailand	2007	CLSI (NCCLS)	2006				(149)

First author	Manuscript title	Publication year	Stated AST guideline	Guideline version	Target organism	Target antimicrobial	Problem identified	Ref.
Sudhalkar A	*Pantoea agglomerans* endophthalmitis: clinical features and outcomes	2014						(150)
Takpere AY	Bacterial isolates, risk factors and antibiogram of neonatal septicemia	2014						(151)
Talawadekar NN	Chloramphenicol resistant *Salmonella* species isolated between 1978 and 1987	1989						(152)
Tankhiwale SS	An unusually high occurrence of *Salmonella enterica* serotype Paratyphi A in patients with enteric fever	2003						(153)
Tarai B	*Hemophilus influenzae* meningitis and septicaemia in a 14-month-old child after primary immunisation	2015	CLSI (NCCLS)	2012				(154)
Thananki RB	"Danger in the blood" BSI and current trend in antimicrobial resistance	2014			*Klebsiella pneumoniae*	Ampicillin	Reports susceptible isolates	(155)
					Staphylococcus aureus	Beta-lactams	Reports ceftazidime	
Thomas K	Invasive pneumococcal disease associated with high case fatality in India	2013	CLSI (NCCLS)	2008				(156)
Threlfall EJ	Widespread occurrence of multiple drug-resistant *Salmonella typhi* in India	1992						(157)
Tiwari DK	A study on the bacteriological profile and antibiogram of bacteremia in children below 10 years in a tertiary care hospital in Bangalore, India	2013	CLSI (NCCLS)	2012	*Salmonellae*	Gentamicin	Reports result (and susceptible isolates)	(158)
					Staphylococcus aureus	Beta-lactams	None	
Tiwari P	Profile and sensitivity pattern of bacteria isolated from various cultures in a Tertiary Care Hospital in Delhi	2010			*Staphylococcus aureus*	Beta-lactams	None	(159)
Tjoa E	*Acinetobacter baumannii*: role in blood stream infection in Neonatal Unit, Dr Cipto Mangunkusumo Hospital, Jakarta, Indonesia	2013	CLSI (NCCLS)	2010				(160)
Treebupachatsakul P	Brain abscess due to *Listeria monocytogenes*: first case report in Thailand	2006	CLSI (NCCLS)	2004				(161)
Tsering DC	Bacteriological profile of septicemia and the risk factors in neonates and infants in Sikkim	2011	CLSI (NCCLS)	Not stated	*Salmonellae*	Gentamicin	Reports result	(162)
					Staphylococcus aureus	Beta-lactams	None	
Turner P	*Aeromonas* spp bacteremia in pregnant women, Thailand-Myanmar border, 2011	2012	CLSI (NCCLS)	2010				(163)
Valsalan R	Melioidosis masquerading as enteric fever	2008						(164)

First author	Manuscript title	Publication year	Stated AST guideline	Guideline version	Target organism	Target antimicrobial	Problem identified	Ref.
Vijayan AP	*Chromobacterium violaceum* sepsis in an infant	2009						(165)
Visudhiphan P	*Salmonella* meningitis in Thai infants: clinical case reports	1998						(166)
Viswanathan R	Profile of neonatal septicaemia at a district-level sick newborn care unit	2012	CLSI (NCCLS)	2008				(167)
Viswanathan R	Multi-drug resistant gram negative bacilli causing early neonatal sepsis in India	2012	CLSI (NCCLS)	2008				(168)
Viswanathan R	Multi-drug-resistant, non-fermenting, gram-negative bacilli in neonatal sepsis in Kolkata, India: a 4-year study	2014	CLSI (NCCLS)	2008				(169)
Waheed M	The etiology of neonatal sepsis and patterns of antibiotic resistance	2003			*Staphylococcus aureus*	Beta-lactams	Does not report oxacillin / cefoxitin but reports ceftazidime	(170)
Wain J	Quantitation of bacteria in bone marrow from patients with typhoid fever: relationship between counts and clinical features	2001	Not stated					(171)
Walia M	Current perspectives of enteric fever: a hospital-based study from India	2005	CLSI (NCCLS)	2000				(172)
Williams EJ	Hospital-based surveillance of invasive pneumococcal disease among young children in urban Nepal	2009	CLSI (NCCLS)	2006	*Streptococcus pneumoniae*	Penicillin	None	(173)
Win MM	Pneumococcal infection in children attending Yangon Children's Hospital	2014	Not stated		*Streptococcus pneumoniae*	Gentamicin / Penicillin	Reports isolates susceptible to gentamicin and penicillin non-susceptibility on basis of disk testing only	(174)
Zakariya BP	Neonatal sepsis in a tertiary care hospital in South India: bacteriological profile and antibiotic sensitivity pattern	2011	CLSI (NCCLS)	2006	*Streptococcus pneumoniae*	Oxacillin	None	(175)
Zaw Than H	Detection of *Burkholderia pseudomallei* in patients with suppurative infections attending the Yangon General Hospital and New Yangon General Hospital	2013						(176)
Zubair M	Incidence of coagulase negative staphylococci in neonatal sepsis	2011	CLSI (NCCLS)	2010				(177)

References

1. Abdullah FE, Haider F, Fatima K, Irfan S, Iqbal MS. Enteric fever in Karachi: current antibiotic susceptibility of *Salmonellae* isolates. J Coll Physicians Surg Pak. 2012;22(3):147-50.
2. Achana V, Silpapojakul K, Thininta W, Kalnaowakul S. Acute *Pseudomonas pseudomallei* pneumonia and septicemia following aspiration of contaminated water: a case report. Southeast Asian J Trop Med Public Health. 1985;16(3):500-4.
3. Acharya D, Trakulsomboon S, Madhup SK, Korbsrisate S. Antibiotic susceptibility pattern and the indicator of decreased ciprofloxacin susceptibility of *Salmonella enterica* serovar Typhi isolated from Dhulikhel Hospital, Nepal. Jpn J Infect Dis. 2012;65(3):264-7.
4. Agnihotri N, Kaistha N, Gupta V. Antimicrobial susceptibility of isolates from neonatal septicemia. Jpn J Infect Dis. 2004;57(6):273-5.
5. Al-Taiar A, Hammoud MS, Cuiqing L, Lee JK, Lui KM, Nakwan N, et al. Neonatal infections in China, Malaysia, Hong Kong and Thailand. Arch Dis Child Fetal Neonatal Ed. 2013;98(3):F249-55.
6. Amatya NM, Shrestha B, Lekhak B. Etiological agents of bacteraemia and antibiotic susceptibility pattern in Kathmandu Model Hospital. J Nepal Med Assoc. 2007;46(167):112-8.
7. Anand AC. The anatomy of an epidemic (the final report on an epidemic of multidrug resistant enteric fever in eastern India). Trop Gastroenterol. 1993;14(1):21-7.
8. Anderson M, Luangxay K, Sisouk K, Vorlasan L, Soumphonphakdy B, Sengmouang V, et al. Epidemiology of bacteremia in young hospitalized infants in Vientiane, Laos, 2000-2011. J Trop Pediatr. 2014;60(1):10-6.
9. Anekthananon T, Ratanasuwan W, Techasathit W, Komolpis P, Dhiraputra C, Suwanagool S. Community-acquired *Klebsiella* bacteremia. J Infect Dis Antimicrob Agents. 2001;18:15-8.
10. Anjum P, Qureshi AH, Rafi S. Fluroquinolone resistance in typhoidal *Salmonella* and its detection by nalidixic acid disc diffusion. J Pak Med Assoc. 2004;54(6):295-301.
11. Antony B, Pinto H, Dias M, Shetty AK, Scaria B, Kuruvilla T, et al. Spectrum of melioidosis in the suburbs of Mangalore, S west coast of India. Southeast Asian J Trop Med Public Health. 2010;41(1):169-74.
12. Anunnatsiri S, Chansung K, Chetchotisakd P, Sirijerachai C. Febrile neutropenia: a retrospective study in Srinagarind Hospital. J Infect Dis Antimicrob Agents. 1998;15:115-22.
13. Arafat MY, Sobur MA, Haq MA, Islam MN. Current pattern in antimicrobial susceptibility in enteric fever in a private medical college. Bangladesh J Med Sci. 2014;13:67-9.
14. Arifeen SE, Saha SK, Rahman S, Rahman KM, Rahman SM, Bari S, et al. Invasive pneumococcal disease among children in rural Bangladesh: results from a population-based surveillance. Clin Infect Dis. 2009;48 Suppl 2:S103-13.
15. Ariffin H, Ariffin W, Peng LH, Parasakthi N. Septicaemia in paediatric cancer patients: a 5-year surveillance study in university hospital, Kuala Lumpur, Malaysia. J Trop Pediatr. 1997;43(5):279-81.
16. Arora RK, Gupta A, Joshi NM, Kataria VK, Lall P, Anand AC. Multidrug resistant typhoid fever: study of an outbreak in Calcutta. Indian Pediatr. 1992;29(1):61-6.
17. Arora S, Gautam V, Ray P. Changing susceptibility patterns of nonfermenting Gram-negative bacilli. Indian J Med Microbiol. 2012;30(4):485-6.
18. Aurangzeb B, Hameed A. Neonatal sepsis in hospital-born babies: bacterial isolates and antibiotic susceptibility patterns. J Coll Physicians Surg Pak. 2003;13(11):629-32.
19. Ayyagari A, Pal N. Outbreak of typhoid fever due to multiresistant *Salmonella typhi* in northern India - a preliminary report. Trans R Soc Trop Med Hyg. 1991;85:302.
20. Bajracharya BL, Baral MR, Shakya S, Tuladhar P, Paudel M, Acharya B. Clinical profile and antibiotics response in typhoid fever. Kathmandu Univ Med J. 2006;4(1):25-9.

21. Baker S, Campbell JI, Stabler R, Nguyen HV, To DS, Nguyen DV, et al. Fatal wound infection caused by *Chromobacterium violaceum* in Ho Chi Minh City, Vietnam. J Clin Microbiol. 2008;46(11):3853-5.
22. Banerjee M, Sahu K, Bhattacharya S, Adhya S, Bhowmick P, Chakraborty P. Outbreak of neonatal septicemia with multidrug resistant *Klebsiella pneumoniae*. Indian J Ped. 1993;60:25-7.
23. Bankar S, De A, Baveja S. Study of ascitic fluid for diagnosis of Spontaneous Bacterial Peritonitis (SBP) in adult patients with cirrhosis. Int J Med Appl Sci. 2014;3:1-9.
24. Baskaran ND, Gan GG, Adeeba K, Sam IC. Bacteremia in patients with febrile neutropenia after chemotherapy at a university medical center in Malaysia. Int J Infect Dis. 2007;11(6):513-7.
25. Behera B, Prasad Babu TL, Kamalesh A, Reddy G. Ceftazidime resistance in *Burkholderia pseudomallei*: first report from India. Asian Pac J Trop Med. 2012;5(4):329-30.
26. Bhagawati G, Barkataki D, Hazarika NK. Study on isolates of acute meningitis in a tertiary care centre in Assam. Int J Med Public Health. 2014;4:446-50.
27. Bhagra S, Kanga A, Ganju SA, Sood A. Antibiotic susceptibility pattern of *Salmonella enterica* serovar Typhi and Paratyphi a from North India: The changing scenario. Int J Pharma Bio Sci. 2014;5:B1-B9.
28. Bhaskar MM, Harish BN. Changing paradigm of enterococcal infections. Int J Pharma Bio Sci. 2015;6:349-53.
29. Bhat KG, Paul C, Bhat MG. Neonatal bacteremia due to high level aminoglycoside resistant (HLAR) enterococci. Indian J Pediatr. 1997;64(4):537-9.
30. Bhattacharya SS, Das U. Occurrence of *Salmonella typhi* infection in Rourkela, Orissa. Indian J Med Res. 2000;111:75-6.
31. Bhattacharya SS, Das U, Choudhury BK. Occurrence & antibiogram of *Salmonella* Typhi & *S*. Paratyphi A isolated from Rourkela, Orissa. Indian J Med Res. 2011;133:431-3.
32. Bindu D, Chitralekha S, Menezes GA, Illamani V. Bacterial profile and antibiotic susceptibility pattern of blood culture isolates from pediatric age group attending a tertiary care centre. Res J Pharm Biol Chem Sci. 2013;4:299-303.
33. Boonyagars L, Chongtrakool P, Watcharananan SP. Meningitis and spondylodiscitis due to S*treptococcus suis*. J Infect Dis Antimicrob Agents. 2010;27:129-33.
34. Butt T, Afzal RK, Ahmad RN, Hussain I, Anwar M. Central venous catherter-related bloodstream infections in cancer patients. J Coll Physicians Surg Pak. 2004;14:549-52.
35. Butt T, Ahmad RN, Salman M, Kazmi SY. Changing trends in drug resistance among typhoid *salmonellae* in Rawalpindi, Pakistan. East Mediterr Health J. 2005;11(5-6):1038-44.
36. Capoor MR, Nair D, Aggarwal P, Mathys V, Dehem M, Bifani PJ. *Salmonella enterica* serovar typhi: molecular analysis of strains with decreased susceptibility and resistant to ciprofloxacin in India from 2001-2003. Braz J Infect Dis. 2007;11(4):423-5.
37. Chaikittisuk N. *Salmonella* bacteremia in children: a changing pattern of antibiotic resistance. J Infect Dis Antimicrob Agents. 2000;17:23-7.
38. Chande C, Shrikhande S, Kapale S, Agrawal S, Fule RP. Change in antimicrobial resistance pattern of *Salmonella* Typhi in central India. Indian J Med Res. 2002;115:248-50.
39. Chim H, Song C. *Aeromonas* infection in critically ill burn patients. Burns. 2007;33(6):756-9.
40. Chlebicki MP, Ling ML, Koh TH, Hsu LY, Tan BH, How KB, et al. First outbreak of colonization and infection with vancomycin-resistant *Enterococcus faecium* in a tertiary care hospital in Singapore. Infect Control Hosp Epidemiol. 2006;27(9):991-3.
41. Chong CY, Koh-Cheng T, Yee-Hui M, Nancy TW. Invasive pneumococcal disease in Singapore children. Vaccine. 2008;26(27-28):3427-31.

42. Choudhary A, Gopalakrishnan R, Nambi PS, Ramasubramanian V, Ghafur KA, Thirunarayan MA. Antimicrobial susceptibility of *Salmonella enterica* serovars in a tertiary care hospital in southern India. Indian J Med Res. 2013;137(4):800-2.
43. Deris ZZ, Harun A, Omar M, Johari MR. The prevalence and risk factors of nosocomial *Acinetobacter* blood stream infections in tertiary teaching hospital in north-eastern Malaysia. Trop Biomed. 2009;26(2):123-9, 219-22.
44. Dhanashree B. Antibiotic susceptibility profile of *Salmonella enterica* serovars: trend over three years showing re-emergence of chloramphenicol sensitivity and rare serovars. Indian J Med Sci. 2007;61(10):576-9.
45. Duggal S, Duggal N, Charoo H, Mahajan RK. Recent outbreak of meningococcal meningitis--a microbiological study with brief review of literature. J Commun Dis. 2007;39(4):209-16.
46. Duggal S, Rongpharpi SR, Gur R, Nayar R, Arora VM. Etiology and susceptibility of blood stream infections in a Referral Hospital in North Delhi: A one year study. Res J Pharm Biol Chem Sci. 2014;5:1859-64.
47. Dutta S, Sur D, Manna B, Bhattacharya SK, Deen JL, Clemens JD. Rollback of *Salmonella enterica* serotype Typhi resistance to chloramphenicol and other antimicrobials in Kolkata, India. Antimicrob Agents Chemother. 2005;49(4):1662-3.
48. Easow JM, Joseph NM, Dhungel BA, Chapagain B, Shivananda PG. Blood stream infections among febrile patients attending a teaching hospital in Western Region of Nepal. Australas Med J. 2010;3:633-7.
49. Emary K, Moore CE, Chanpheaktra N, An KP, Chheng K, Sona S, et al. Enteric fever in Cambodian children is dominated by multidrug-resistant H58 *Salmonella enterica* serovar Typhi with intermediate susceptibility to ciprofloxacin. Trans R Soc Trop Med Hyg. 2012;106(12):718-24.
50. Geetha VK, Yugendran T, Srinivasan R, Harish BN. Plasmid-mediated quinolone resistance in typhoidal *Salmonellae*: a preliminary report from South India. Indian J Med Microbiol. 2014;32(1):31-4.
51. Geethu M, Prabhu N, Jasmine MK. Antimicrobial susceptibility trends among viridans streptococci isolates from cases of endocarditis from 2007-2009. Ann Biol Res. 2010;1:130-3.
52. Gopal Katherason S, Naing L, Jaalam K, Kamarul Iman Musa K, Nik Abdullah NM, Aiyar S, et al. Prospective surveillance of nosocomial device-associated bacteremia in three adult intensive units in Malaysia. Trop Biomed. 2010;27(2):308-16.
53. Gupta P, Murali MV, Faridi MM, Kaul PB, Ramachandran VG, Talwar V. Clinical profile of *Klebsiella* septicemia in neonates. Indian J Pediatr. 1993;60(4):565-72.
54. Gupta V, Kaur J, Chander J. An increase in enteric fever cases due to *Salmonella* Paratyphi A in & around Chandigarh. Indian J Med Res. 2009;129(1):95-8.
55. Gyawali N, Sanjana RK. Bacteriological profile and antibiogram of neonatal septicemia. Indian J Ped. 2013;80(5):371-4.
56. Habib AG, Tambyah PA. Community-acquired *Klebsiella pneumoniae* central nervous system infections in adults in Singapore. Eur J Clin Microbiol Infect Dis. 2003;22(8):486-8.
57. Halder D, Zainal N, Wah CM, Haq JA. Neonatal meningitis and septicaemia caused by *Burkholderia pseudomallei*. Ann Trop Paediatr. 2016;18(2):161-4.
58. Haque SM, Jahan N, Mannan MA, Hasan M, Begum M, Rob S, et al. Identification of bacterial isolates in neonatal sepsis and their antimicrobial susceptibility. Mymensingh Med J. 2014;23(4):709-14.
59. Heng BH, Goh KT, Yap EH, Loh H, Yeo M. Epidemiological surveillance of melioidosis in Singapore. Ann Acad Med Singapore. 1998;27(4):478-84.
60. Hoa NT, Diep TS, Wain J, Parry CM, Hien TT, Smith MD, et al. Community-acquired septicaemia in southern Viet Nam: the importance of multidrug-resistant *Salmonella typhi*. Trans R Soc Trop Med Hyg. 1998;92(5):503-8.

61. Hsu LY, Leong M, Balm M, Chan DS, Huggan P, Tan TY, et al. Six cases of daptomycin-non-susceptible *Staphylococcus aureus* bacteraemia in Singapore. J Med Microbiol. 2010;59(Pt 12):1509-13.
62. Imran MN, Leng PH, Yang S, Kurup A, Eng P. Early predictors of mortality in pneumococcal bacteraemia. Ann Acad Med Singapore. 2005;34(7):426-31.
63. Iqbal Hossain M, Iqbal Kabir AKM, Khan WA, Fuchs GJ. *Acinetobacter* bacteraemia in patients with diarrhoeal disease. Epidemiol Infect. 1998;120:139-42.
64. Jain N, Mathur P, Misra MC. *Globicatella sanguinis* meningitis in a post head trauma patient: first case report from Asia. J Infect Dev Ctries. 2012;6(7):592-4.
65. Jesudason MV, Anbarasu A, John TJ. Septicaemic melioidosis in a tertiary care hospital in south India. Indian J Med Res. 2003;117:119-21.
66. Jitsurong S, Yodsawat J. Prevalence of extended-spectrum beta-lactamases (ESBLs) produced in blood isolates of gram-negative bacteria in a teaching hospital in southern Thailand. Southeast Asian J Trop Med Public Health. 2006;37(1):131-5.
67. Kabra SK, Madhulika, Talati A, Soni N, Patel S, Modi RR. Multidrug-resistant typhoid fever. Trop Doct. 2000;30(4):195-7.
68. Kamili MA, Ali G, Shah MY, Rashid S, Khan S, Allaqaband GQ. Multiple drug resistant typhoid fever outbreak in Kashmir Valley. Indian J Med Sci. 1993;47(6):147-51.
69. Kapoor L, Randhawa VS, Deb M. Microbiological profile of neonatal septicemia in a pediatric care hospital in Delhi. J Commun Dis. 2005;37(3):227-32.
70. Karki AB, Bhatta DR, Shrestha B, Khatri B, Rai KR, Mukhiya RK, et al. Higher nalidixic acid resistance pattern of *Salmonella* isolates from enteric fever patients in Kathmandu Model Hospital Nepal. Res J Pharm Biol Chem Sci. 2013;4:1687-93.
71. Karki S, Manandhar R. Bacteriological Analysis and Antibiotic Sensitivity Pattern of Blood Culture Isolates in Kanti Children Hospital. J Nepal Paediatr Soc. 2010;30:94-7.
72. Karthikeyan G, Premkumar K. Neonatal sepsis: *Staphylococcus aureus* as the predominant pathogen. Indian J Pediatr. 2001;68(8):715-7.
73. Kasper MR, Sokhal B, Blair PJ, Wierzba TF, Putnam SD. Emergence of multidrug-resistant *Salmonella enterica* serovar Typhi with reduced susceptibility to fluoroquinolones in Cambodia. Diagn Microbiol Infect Dis. 2010;66(2):207-9.
74. Khairuddin NYN, Choo KE, Johari MR. Epidemiology of *Haemophilus influenzae* invasive disease in hospitalised Kelantanese children, 1985-1994. Singapore Med J. 1999;40:96-100.
75. Khan MI, Ochiai RL, von Seidlein L, Dong B, Bhattacharya SK, Agtini MD, et al. Non-typhoidal *Salmonella* rates in febrile children at sites in five Asian countries. Trop Med Int Health. 2010;15(8):960-3.
76. Komolpis P, Srifuengfung S, Dhiraputra C, Pingwang B. *Salmonella* bacteremia: serotype distribution and antimicrobial susceptibility during 1991-1995. J Infect Dis Antimicrob Agents. 1999;16:49-52.
77. Kruse AY, Thieu Chuong Do H, Phuong CN, Duc T, Graff Stensballe L, Prag J, et al. Neonatal bloodstream infections in a pediatric hospital in Vietnam: a cohort study. J Trop Pediatr. 2013;59(6):483-8.
78. Kumar A, Nath G, Bhatia BD, Bhargava V, Loiwal V. An outbreak of multidrug resistant *Salmonella typhimurium* in a nursery. Indian Pediatr. 1995;32(8):881-5.
79. Kumarasamy K, Krishnan P. Report of a *Salmonella enterica* serovar Typhi isolate from India producing CMY-2 AmpC beta-lactamase. J Antimicrob Chemother. 2012;67(3):775-6.
80. Lee WS, Puthucheary SD, Parasakthi N, Choo KE. Antimicrobial susceptibility and distribution of non-typhoidal *Salmonella* serovars isolated in Malaysian children. J Trop Pediatr. 2003;49(1):37-41.
81. Lim NL, Wong YH, Boo NY, Kasim MS, Chor CY. Bacteraemic infections in a neonatal intensive care unit--a nine-month survey. Med J Malaysia. 1995;50(1):59-63.

82. Litzow JM, Gill CJ, Mantaring JBV, Fox M, Mendoza M, Mendoza S, et al. High frequency of multi drug resistant Gram-negative rods in two neonatal intensive care units in the Philippines. Infect Control Hosp Epidemiol. 2009;30.
83. Lulitanond A, Kanyota R, Engchanil C, Chanawong A, Wilailuckana C, Tavichakorntrakool R, et al. Virulence genes and genotypes of *Staphylococcus aureus* from blood of Thai patients. ScienceAsia. 2015;41:162-9.
84. Lupisan SP, Herva E, Sombrero LT, Quiambao BP, Capeding MR, Abucejo PE, et al. Invasive bacterial infections of children in a rural province in the central Philippines. Am J Trop Med Hyg. 2000;62(3):341-6.
85. Manchanda V, Bhalla P, Sethi M, Sharma VK. Treatment of enteric fever in children on the basis of current trends of antimicrobial susceptibility of *Salmonella enterica* serovar Typhi and Paratyphi A. Indian J Med Microbiol. 2006;24(2):101-6.
86. Maskey AP, Day JN, Phung QT, Thwaites GE, Campbell JI, Zimmerman M, et al. *Salmonella enterica* serovar Paratyphi A and *S. enterica* serovar Typhi cause indistinguishable clinical syndromes in Kathmandu, Nepal. Clin Infect Dis. 2006;42(9):1247-53.
87. Maskey AP, Basnyat B, Thwaites GE, Campbell JI, Farrar JJ, Zimmerman MD. Emerging trends in enteric fever in Nepal: 9124 cases confirmed by blood culture 1993-2003. Trans R Soc Trop Med Hyg. 2008;102(1):91-5.
88. Mehar V, Yadav D, Somani P, Bhatambare G, Mulye S, Singh K. Neonatal sepsis in a tertiary care center in central India: microbiological profile, antimicrobial sensitivity pattern and outcome. J Neonatal Perinatal Med. 2013;6(2):165-72.
89. Mehar V, Yadav D, Sanghvi J, Gupta N, Singh K. *Pantoea dispersa*: an unusual cause of neonatal sepsis. Braz J Infect Dis. 2013;17(6):726-8.
90. Mishra S, Patwari AK, Anand VK, Pillai PK, Aneja S, Chandra J, et al. Multidrug resistant typhoid fever: therapeutic considerations. Indian Pediatr. 1992;29(4):443-8.
91. Mohanty S, Renuka K, Sood S, Das BK, Kapil A. Antibiogram pattern and seasonality of *Salmonella* serotypes in a North Indian tertiary care hospital. Epidemiol Infect. 2006;134(5):961-6.
92. Mohanty S, Kapil A, Das BK. Bacteriology of parapneumonic pleural effusions in an Indian hospital. Trop Doct. 2007;37(4):228-9.
93. Mortlock S. Bacteraemia among patients attending a cancer hospital in Lahore, Pakistan. Br J Biomed Sci. 2000;57(2):119-25.
94. Muhammad Z, Ahmed A, Hayat U, Wazir MS, Waqas H, Zardad M, et al. Neonatal sepsis: causative bacteria and their resistance to antibiotics. J Ayub Med Coll Abbottabad. 2010;22:33-6.
95. Muley VA, Ghadage DP, Bhore AV. Bacteriological profile of neonatal septicemia in a tertiary care hospital from Western India. J Glob Infect Dis. 2015;7(2):75-7.
96. Murdoch DR, Woods CW, Zimmerman MD, Dull PM, Belbase RH, Keenan AJ, et al. The etiology of febrile illness in adults presenting to Patan hospital in Kathmandu, Nepal. Am J Trop Med Hyg. 2004;70(6):670-5.
97. Naheed A, Ram PK, Brooks WA, Hossain MA, Parsons MB, Talukder KA, et al. Burden of typhoid and paratyphoid fever in a densely populated urban community, Dhaka, Bangladesh. Int J Infect Dis. 2010;14 Suppl 3:e93-9.
98. Najeeb S, Gillani S, Rizvi SK, Ullah R, ur Rehman A. Causative bacteria and antibiotic resistance in neonatal sepsis. J Ayub Med Coll Abbottabad. 2012;24(3-4):131-4.
99. Nakwan N, Wannaro J, Nakwan N, Patungkalo W, Chokephaibulkit K. Clinical features, risk factors, and outcome of carbapenem-resistant *Acinetobacter baumannii* bacteremia in a Thai neonatal intensive care unit. Asian Biomedicine. 2012;6:473-9.

100. Nandhakumar B, Senthilkumar S, Menon T, Shanmugasundaram S. Penicillin-resistant viridans group streptococci from blood cultures of infective endocarditis patients in South India. Int J Antimicrob Agents. 2008;32(6):543-4.
101. Nema S, Chitnis DS. Antibiogram study over bacterial isolates from cases of bacteraemias. Indian J Med Sci. 1996;50(9):325-9.
102. Neopane A, Singh SB, Bhatta R, Dhital B, Karki DB. Changing spectrum of antibiotic sensitivity in enteric fever. Kathmandu Univ Med J. 2008;6(1):12-5.
103. Nickerson EK, Hongsuwan M, Limmathurotsakul D, Wuthiekanun V, Shah KR, Srisomang P, et al. *Staphylococcus aureus* bacteraemia in a tropical setting: patient outcome and impact of antibiotic resistance. PLoS One. 2009;4(1):e4308.
104. Ochiai RL, Acosta CJ, Danovaro-Holliday MC, Baiqing D, Bhattacharya SK, Agtini MD, et al. A study of typhoid fever in five Asian countries: disease burden and implications for controls. Bull World Health Organ. 2008;86(4):260-8.
105. Ong CW, Lye DC, Khoo KL, Chua GS, Yeoh SF, Leo YS, et al. Severe community-acquired *Acinetobacter baumannii* pneumonia: an emerging highly lethal infectious disease in the Asia-Pacific. Respirology. 2009;14(8):1200-5.
106. Pais M, Devi ES, Pai MV, Lewis L, Gorge A, Mayya SS, et al. Neonatal sepsis, bacterial isolates and antibiotic susceptibility patterns among neonates. Nursing J India. 2012;103(1):18-20.
107. Palash S, Vikas G, Rajni T, Pallab R. Emerging resistance of non-fermenting gram negative bacilli in a tertiary care centre. Indian J Pathol Microbiol. 2011;54:666-7.
108. Pancharoen C, Thisyakorn U. Meningococcal infection in children. J Infect Dis Antimicrob Agents. 1998:55-8.
109. Pandit V, Kumar A, Kulkarni MM, Pattanshetty SM, Samarasinghe C, Kamath S. Study of clinical profile and antibiotic sensitivity in paratyphoid fever cases admitted at teaching hospital in South India. J Family Med Prim Care. 2012;1(2):118-21.
110. Parveen RM, Khan MA, Menezes GA, Harish BN, Parija SC, Hays JP. Extended-spectrum β-lactamase producing *Klebsiella pneumoniae* from blood cultures in Puducherry, India. Indian J Med Res. 2011;134:392-5.
111. Pathengay A, Moreker MR, Puthussery R, Ambatipudi S, Jalali S, Majji AB, et al. Clinical and microbiologic review of culture-proven endophthalmitis caused by multidrug-resistant bacteria in patients seen at a tertiary eye care center in southern India. Retina. 2011;31(9):1806-11.
112. Phan LT, Ngo TT, Dang DA, Vu TT, Le NM, Tran QC, et al. Genetic and phenotypic characterization of *Haemophilus influenzae* type b isolated from children with meningitis and their family members in Vietnam. Jpn J Infect Dis. 2006;59(2):111-6.
113. Phe T, Vlieghe E, Reid T, Harries AD, Lim K, Thai S, et al. Does HIV status affect the aetiology, bacterial resistance patterns and recommended empiric antibiotic treatment in adult patients with bloodstream infection in Cambodia? Trop Med Int Health. 2013;18(4):485-94.
114. Phetsouvanh R, Phongmany S, Soukaloun D, Rasachak B, Soukhaseum V, Soukhaseum S, et al. Causes of community-acquired bacteremia and patterns of antimicrobial resistance in Vientiane, Laos. Am J Trop Med Hyg. 2006;75(5):978-85.
115. Pocock JM, Khun PA, Moore CE, Vuthy S, Stoesser N, Parry CM. Septic arthritis of the hip in a Cambodian child caused by multidrug-resistant *Salmonella enterica* serovar Typhi with intermediate susceptibility to ciprofloxacin treated with ceftriaxone and azithromycin. Paediatr Int Child Health. 2014;34(3):227-9.
116. Pokharel BM, Koirala J, Dahal RK, Mishra SK, Khadga PK, Tuladhar NR. Multidrug-resistant and extended-spectrum beta-lactamase (ESBL)-producing *Salmonella enterica* (serotypes Typhi and Paratyphi A) from blood isolates in Nepal: surveillance of resistance and a search for newer alternatives. Int J Infect Dis. 2006;10(6):434-8.

117. Pokharel P, Rai SK, Karki G, Katuwal A, Vitrakoti R, Shrestha SK. Study of enteric fever and antibiogram of *Salmonella* isolates at a teaching hospital in Kathmandu Valley. Nepal Med Coll J. 2009;11(3):176-8.
118. Porter KA, Rhodes J, Dejsirilert S, Henchaichon S, Siludjai D, Thamthitiwat S, et al. *Acinetobacter* bacteraemia in Thailand: evidence for infections outside the hospital setting. Epidemiol Infect. 2014;142(6):1317-27.
119. Pradhan R, Shrestha U, Gautam SC, Thorson S, Shrestha K, Yadav BK, et al. Bloodstream infection among children presenting to a general hospital outpatient clinic in urban Nepal. PLoS One. 2012;7(10):e47531.
120. Punpanich W, Netsawang S, Thippated C. Invasive salmonellosis in urban Thai children: a ten-year review. Pediatr Infect Dis J. 2012;31(8):e105-10.
121. Qamar FN, Azmatullah A, Kazi AM, Khan E, Zaidi AK. A three-year review of antimicrobial resistance of *Salmonella enterica* serovars Typhi and Paratyphi A in Pakistan. J Infect Dev Ctries. 2014;8(8):981-6.
122. Qamar MU, Abdul H, Arshad MU, Muhammad A. Metallo-beta-lactamase producing *Enterobacter cloacae*: an emerging threat in neonates. Afr J Microbiol Res. 2014;8:525-8.
123. Rai S, Jain S, Prasad KN, Ghoshal U, Dhole TN. Rationale of azithromycin prescribing practices for enteric fever in India. Indian J Med Microbiol. 2012;30(1):30-3.
124. Rao RS, Sundararaj T, Subramanian S, Shankar V, Murty SA, Kapoor SC. A study of drug resistance among *Salmonella typhi* and Salmonella *paratyphi A* in an endemic area, 1977-79. Trans R Soc Trop Med Hyg. 1981;75(1):21-4.
125. Raza S, Tamrakar R, Bhatt CP, Joshi SK. Antimicrobial susceptibility patterns of *Salmonella typhi* and *Salmonella paratyphi A* in a tertiary care hospital. J Nepal Health Res Counc. 2012;10(22):214-7.
126. Renuka K, Kapil A, Kabra SK, Wig N, Das BK, Prasad VV, et al. Reduced susceptibility to ciprofloxacin and gyra gene mutation in North Indian strains of *Salmonella enterica* serotype Typhi and serotype Paratyphi A. Microb Drug Resist. 2004;10(2):146-53.
127. Rhodes J, Dejsirilert S, Maloney SA, Jorakate P, Kaewpan A, Salika P, et al. Pneumococcal bacteremia requiring hospitalization in rural Thailand: an update on incidence, clinical characteristics, serotype distribution, and antimicrobial susceptibility, 2005-2010. PLoS One. 2013;8(6):e66038.
128. Roy S, Datta S, Viswanathan R, Singh AK, Basu S. Tigecycline susceptibility in *Klebsiella pneumoniae* and Escherichia coli causing neonatal septicaemia (2007-10) and role of an efflux pump in tigecycline non-susceptibility. J Antimicrob Chemother. 2013;68(5):1036-42.
129. Sharma NP, Peacock SJ, Phumratanaprapin W, Day N, White N, Pukrittayakamee S. A hospital-based study of bloodstream infections in febrile patients in Dhulikhel Hospital Kathmandu University Teaching Hospital, Nepal. Southeast Asian J Trop Med Public Health. 2006;37(2):351-6.
130. Sharma P, Kaur P, Aggarwal A. *Staphylococcus aureus* - the predominant pathogen in the neonatal ICU of a tertiary care hospital in Amritsar, India. J Clin Diagn Res. 2013;7(1):66-9.
131. Shaw P, Shaw CK, Saileela K. Microbial array and antibiotic sensitivity pattern of catheter related blood-stream infection at a tertiary care hospital in South India. Int J Pharma Bio Sci. 2012;3:100-7.
132. Sheikh A, Bhuiyan MS, Khanam F, Chowdhury F, Saha A, Ahmed D, et al. *Salmonella enterica* serovar Typhi-specific immunoglobulin A antibody responses in plasma and antibody in lymphocyte supernatant specimens in Bangladeshi patients with suspected typhoid fever. Clin Vaccine Immunol. 2009;16(11):1587-94.
133. Sheikh AN, Sajjad A, Hanif S. Neonatal sepsis: An evaluation of bacteriological spectrum, antibiotic susceptibilities and prognostic predictors at Civil Hospital, Karachi. Pak Paed J. 2014;38:143-55.

134. Sheikh SO, Jabeen K, Qaiser S, Ahsan ST, Khan E, Zafar A. High rate of non-susceptibility to metronidazole and clindamycin in anaerobic isolates: data from a clinical laboratory from Karachi, Pakistan. Anaerobe. 2015;33:132-6.
135. Shenoy S, Vandana KE. Penicillin Resistant *Streptococcus pneumoniae*. Indian Pediatr. 2003;40:587-8.
136. Shivaprakasha S, Radhakrishnan K, Kamath P, Karim PMS. Late prosthetic valve endocarditis due to *Cardiobacterium hominis*, an unusual complication. Indian J Med Microbiol. 2007;25(1):64-6.
137. Shivaprakasha S, Radhakrishnan K, Panikar D, Natarajan KU, Shamsul Karim PM. Cerebral artery mycotic aneurysm associated with *Erysipelothrix rhusiopathiae* endocarditis. Infect Dis Clin Pract. 2007;15:400-2.
138. Shoma S, Rahman M, Yasmin M. Rapid detection of *Haemophilus influenzae* type b in Bangladeshi children with pneumonia and meningitis by PCR and analysis of antimicrobial resistance. J Health Popul Nutr. 2001;19(4):268-74.
139. Shrestha RK, Rai SK, Khanal LK, Manda PK. Bacteriological study of neonatal sepsis and antibiotic susceptibility pattern of isolates in Kathmandu, Nepal. Nepal Med Coll J. 2013;15(1):71-3.
140. Shrestha S, Adhikari N, Rai BK, Shreepaili A. Antibiotic resistance pattern of bacterial isolates in neonatal care unit. J Nepal Med Assoc. 2010;50(180):277-81.
141. Shrestha S, Shrestha NC, Dongol Singh S, Shrestha RPB, Kayestha S, Shrestha M, et al. Bacterial isolates and its antibiotic susceptibility pattern in NICU. Kathmandu Univ Med J. 2013;11:66-70.
142. Shwe TN, Nyein MM, Yi W, Mon A. Blood culture isolates from children admitted to Medical Unit III, Yangon Children's Hospital, 1998. Southeast Asian J Trop Med Public Health. 2002;33(4):764-71.
143. Singh DS, Shrestha S, Shrestha N, Manandhar S. Enteric fever in children at Dhulikhel hospital. J Nepal Paediatr Soc. 2012;32:216-20.
144. Singh UK, Neopane AK, Thapa M, Aryal N, Agrawal K. *Salmonella typhi* infections and effect of fluroquinolones and third generation cephalosporins in clinical outcome. J Nepal Paediatr Soc. 2011;31:216-21.
145. Singhal L, Gupta PK, Kale P, Gautam V, Ray P. Trends in antimicrobial susceptibility of *Salmonella* Typhi from North India (2001-2012). Indian J Med Microbiol. 2014;32(2):149-52.
146. Singhi S, Ray P, Mathew JL, Jayashree M, Dhanalakshmi. Nosocomial bloodstream infection in a pediatric intensive care unit. Indian J Pediatr. 2008;75(1):25-30.
147. Singla N, Bansal N, Gupta V, Chander J. Outbreak of *Salmonella* Typhi enteric fever in sub-urban area of North India: a public health perspective. Asian Pac J Trop Med. 2013;6(2):167-8.
148. Srifuengfung S, Chokephaibulkit K, Yungyuen T, Tribuddharat C. *Salmonella* meningitis and antimicrobial susceptibilities. Southeast Asian J Trop Med Public Health. 2005;36(2):312-6.
149. Srifuengfung S, Tharavichitkul P, Pumprueg S, Tribuddharat C. *Roseomonas gilardii* subsp *rosea*, a pink bacterium associated with bacteremia: the first case in Thailand. Southeast Asian J Trop Med Public Health. 2007;38(5):886-91.
150. Sudhalkar A, Majji AB, Chhablani J, Manderwad G. *Pantoea agglomerans* endophthalmitis: clinical features and outcomes. Retina. 2014;34(8):1702-6.
151. Takpere AY, Kamble VS. Bacterial isolates, risk factors and antibiogram of neonatal septicemia. Int J Pharma Bio Sci. 2014;5:788-93.
152. Talawadekar NN, Vadher PJ, Antani DU, Kale VV, Kamat SA. Chloramphenicol resistant *Salmonella* species isolated between 1978 and 1987. J Postgrad Med. 1989;35(2):79-82.
153. Tankhiwale SS, Agrawal G, Jalgaonkar SV. An unusually high occurrence of *Salmonella enterica* serotype Paratyphi A in patients with enteric fever. Indian J Med Res. 2003;117:10-2.
154. Tarai B, Ravishankar N, Vohra P, Das P. *Hemophilus influenzae* meningitis and septicaemia in a 14-month-old child after primary immunisation. Indian J Med Microbiol. 2015;33(1):158-60.

155. Thananki RB, Kumari HP, Subbarayudu S. "Danger in the blood" BSI and current trend in antimicrobial resistance. Int J Pharma Bio Sci. 2014;5:827-34.
156. Thomas K, Mukkai Kesavan L, Veeraraghavan B, Jasmine S, Jude J, Shubankar M, et al. Invasive pneumococcal disease associated with high case fatality in India. J Clin Epidemiol. 2013;66(1):36-43.
157. Threlfall EJ, Ward LR, Rowe B, Raghupathi S, Chandrasekaran V, Vandepitte J, et al. Widespread occurrence of multiple drug-resistant *Salmonella typhi* in India. Eur J Clin Microbiol Infect Dis. 1992;11(11):990-3.
158. Tiwari DK, Golia S, Sangeetha KT, Vasudha CL. A study on the bacteriological profile and antibiogram of bacteremia in children below 10 years in a tertiary care hospital in Bangalore, India. J Clin Diagn Res. 2013;7(12):2732-5.
159. Tiwari P, Kaur S. Profile and sensitivity pattern of bacteria isolated from various cultures in a Tertiary Care Hospital in Delhi. Indian J Public Health. 2010;54(4):213-5.
160. Tjoa E, Moehario LH, Rukmana A, Rohsiswatmo R. *Acinetobacter baumannii*: role in blood stream infection in Neonatal Unit, Dr Cipto Mangunkusumo Hospital, Jakarta, Indonesia. Int J Microbiol. 2013;2013:180763.
161. Treebupachatsakul P, Srifeungfung S, Chayakulkeeree M. Brain abscess due to *Listeria monocytogenes*: first case report in Thailand. J Med Assoc Thai. 2006;89(9):1516-20.
162. Tsering DC, Chanchal L, Pal R, Kar S. Bacteriological profile of septicemia and the risk factors in neonates and infants in Sikkim. J Glob Infect Dis. 2011;3(1):42-5.
163. Turner P, Willemse C, Phakaudom K, Zin TW, Nosten F, McGready R. *Aeromonas* spp. bacteremia in pregnant women, Thailand-Myanmar border, 2011. Emerg Infect Dis. 2012;18(9):1522-3.
164. Valsalan R, Seshadri S, Pandit VR. Melioidosis masquerading as enteric fever. Trans R Soc Trop Med Hyg. 2008;102 Suppl 1:S117-8.
165. Vijayan AP, Anand MR, Remesh P. *Chromobacterium violaceum* Sepsis in an Infant. Indian Pediatr. 2009;46:721-2.
166. Visudhiphan P, Chiemchanya S, Visutibhan A. *Salmonella* meningitis in Thai infants: clinical case reports. Trans R Soc Trop Med Hyg. 1998;92(2):181-4.
167. Viswanathan R, Singh AK, Ghosh C, Dasgupta S, Mukherjee S, Basu S. Profile of neonatal septicaemia at a district-level sick newborn care unit. J Health Popul Nutr. 2012;30(1):41-8.
168. Viswanathan R, Singh AK, Basu S, Chatterjee S, Sardar S, Isaacs D. Multi-drug resistant gram negative bacilli causing early neonatal sepsis in India. Arch Dis Child Fetal Neonatal Ed. 2012;97(3):F182-7.
169. Viswanathan R, Singh AK, Basu S, Chatterjee S, Roy S, Isaacs D. Multi-drug-resistant, non-fermenting, gram-negative bacilli in neonatal sepsis in Kolkata, India: a 4-year study. Paediatr Int Child Health. 2014;34(1):56-9.
170. Waheed M, Laeeq A, Maqbool S. The etiology of neonatal sepsis and patterns of antibiotic resistance. J Coll Physicians Surg Pak. 2003;13(8):449-52.
171. Wain J, Pham VB, Ha V, Nguyen NM, To SD, Walsh AL, et al. Quantitation of bacteria in bone marrow from patients with typhoid fever: relationship between counts and clinical features. J Clin Microbiol. 2001;39(4):1571-6.
172. Walia M, Gaind R, Mehta R, Paul P, Aggarwal P, Kalaivani M. Current perspectives of enteric fever: a hospital-based study from India. Ann Trop Paediatr. 2005;25(3):161-74.
173. Williams EJ, Thorson S, Maskey M, Mahat S, Hamaluba M, Dongol S, et al. Hospital-based surveillance of invasive pneumococcal disease among young children in urban Nepal. Clin Infect Dis. 2009;48 Suppl 2:S114-22.
174. Win MM, Nyein MM, Htwe MM, Mya T, Shwe TT, Han AM, et al. Pneumococcal infection in children attending Yangon Children's Hospital. Myanmar Health Sci Res J. 2014;26:1-5.

175. Zakariya BP, Bhat V, Harish BN, Arun Babu T, Joseph NM. Neonatal sepsis in a tertiary care hospital in South India: bacteriological profile and antibiotic sensitivity pattern. Indian J Pediatr. 2011;78(4):413-7.
176. Htun ZT, Win MM, Hla T, Myat TW, Lin N, Wah TT. Detection of *Burkholderia pseudomallei* in patients with suppurative infections attending the Yangon General Hospital and New Yangon General Hospital. Myanmar Health Sci Res J. 2013;25:114-20.
177. Zubair M, Zafar A, Ejaz H, Hafeez S, Javaid H, Javed A. Incidence of coagulase negative staphylococci in neonatal sepsis. Pak J Med Health Sci. 2011;5:716-9.
